# Design and evaluation of locked nucleic acid-based splice-switching oligonucleotides *in vitro*

**DOI:** 10.1093/nar/gku512

**Published:** 2014-06-16

**Authors:** Takenori Shimo, Keisuke Tachibana, Kiwamu Saito, Tokuyuki Yoshida, Erisa Tomita, Reiko Waki, Tsuyoshi Yamamoto, Takefumi Doi, Takao Inoue, Junji Kawakami, Satoshi Obika

**Affiliations:** 1 Graduate School of Pharmaceutical Sciences, Osaka University, 1–6, Yamadaoka, Suita, Osaka, 565–0871, Japan; 2 Division of Cellular and Gene Therapy Products, National Institute of Health Sciences, 1–18–1 Kamiyoga, Setagaya-ku, Tokyo 158–8501, Japan; 3 Department of Nanobiochemistry, FIRST, Konan University, 7–1–20 Minatojima-minamimachi, Chuo-ku, Kobe 650–0047, Japan; 4 Frontier Institute for Biomolecular Engineering Research (FIBER), Konan University, 7–1–20 Minatojima-minamimachi, Chuo-ku, Kobe 650–0047, Japan

## Abstract

Antisense-mediated modulation of pre-mRNA splicing is an attractive therapeutic strategy for genetic diseases. Currently, there are few examples of modulation of pre-mRNA splicing using locked nucleic acid (LNA) antisense oligonucleotides, and, in particular, no systematic study has addressed the optimal design of LNA-based splice-switching oligonucleotides (LNA SSOs). Here, we designed a series of LNA SSOs complementary to the human dystrophin exon 58 sequence and evaluated their ability to induce exon skipping *in vitro* using reverse transcription-polymerase chain reaction. We demonstrated that the number of LNAs in the SSO sequence and the melting temperature of the SSOs play important roles in inducing exon skipping and seem to be key factors for designing efficient LNA SSOs. LNA SSO length was an important determinant of activity: a 13-mer with six LNA modifications had the highest efficacy, and a 7-mer was the minimal length required to induce exon skipping. Evaluation of exon skipping activity using mismatched LNA/DNA mixmers revealed that 9-mer LNA SSO allowed a better mismatch discrimination. LNA SSOs also induced exon skipping of endogenous human dystrophin in primary human skeletal muscle cells. Taken together, our findings indicate that LNA SSOs are powerful tools for modulating pre-mRNA splicing.

## INTRODUCTION

Alternative pre-mRNA splicing is an essential system for gene expression in eukaryotes that allows the production of various types of proteins from a limited set of genes (1). However, mutations in splice sites cause mis-splicing, which is followed by genetic diseases (2,3,4). To correct these splicing errors, exon skipping by using antisense oligonucleotides (AONs) has been suggested (5,6). These splice-switching oligonucleotides (SSOs) bind to target sequences in pre-mRNA and prevent the interaction of various splicing modulators (7). Thus, SSOs are able to modulate pre-mRNA splicing and repair defective RNA without inducing the RNase H-mediated cleavage of mRNA (8,9).

To enhance the *in vivo* activity of AONs, many artificial nucleic acids have been synthesized to improve nuclease resistance, binding properties, RNase H activity and serum stability (10,11). Locked nucleic acid (LNA) (also known as 2’-*O*,4’-*C*-methylene-bridged nucleic acid (2’,4’-BNA)) is an artificial nucleic acid derivative that was synthesized by us and by Wengel's group independently in the late 1990s (12,13). LNA contains a methylene bridge connecting the 2’-*O* with the 4’-*C* position in the furanose ring, which enables it to form a strictly *N*-type conformation that offers high binding affinity against complementary RNA (14,15,16). LNA also presents enzyme resistance, similar to other nucleic acid derivatives. Given these features, LNA can be used for various gene silencing techniques, such as antisense, short interfering RNA, blocking of microRNA and triplex-forming oligonucleotides. Previous studies also showed that LNA could be used in SSOs (17,18,19,20), and LNA-based SSOs (LNA SSOs) have been shown to be functional *in vivo* in mouse models (21,22).

Recently, SSOs based on 2’-*O*-methyl RNA (2’-OMe) with a full-length phosphorothioate (PS) backbone, phosphorodiamidate morpholino oligomer or 2’-*O*,4’-*C*-ethylene-bridged nucleic acids have been applied to clinical trials for the treatment of genetic diseases, particularly Duchenne muscular dystrophy (DMD) (23,24,25,26,27,28). DMD is a severe muscle-weakening disease that arises from mutations in dystrophin, which links the cytoskeleton to the extracellular matrix of muscle fibers. Mutations in the dystrophin gene lead to premature termination of translation and prevent the synthesis of a functional gene product. SSO-mediated exon skipping in dystrophin pre-mRNA can restore the reading frame and allow the expression of a truncated but functional dystrophin similar to that found in Becker muscular dystrophy patients, who have relatively milder symptoms (29). Thus, modulation of splicing using SSOs is an attractive strategy for the treatment of genetic diseases, such as DMD. However, relatively few studies have used LNA SSOs compared to those using SSOs based on other chemistries.

Methods for designing effective SSOs have recently been developed and provide insight into factors that are critical for SSO activity, including the melting temperature (*T*_m_), guanine-cytosine content and secondary structures or sequence motifs that correspond to splicing signals of the target RNA (30,31). Because LNA oligonucleotides possess high binding affinity to complementary RNA, the SSOs that incorporate LNA are considered as promising tools for inducing exon skipping. However, no systematic study has addressed the optimal design of LNA SSOs. Therefore, in this study, we designed a series of LNA SSOs complementary to the human dystrophin exon 58 sequence, and evaluated their ability to induce exon skipping using reverse transcription-polymerase chain reaction (RT-PCR) and a minigene reporter encompassing exons 57–59 of the human dystrophin gene.

## MATERIALS AND METHODS

### Synthesis of oligonucleotides

**Figure 1.**
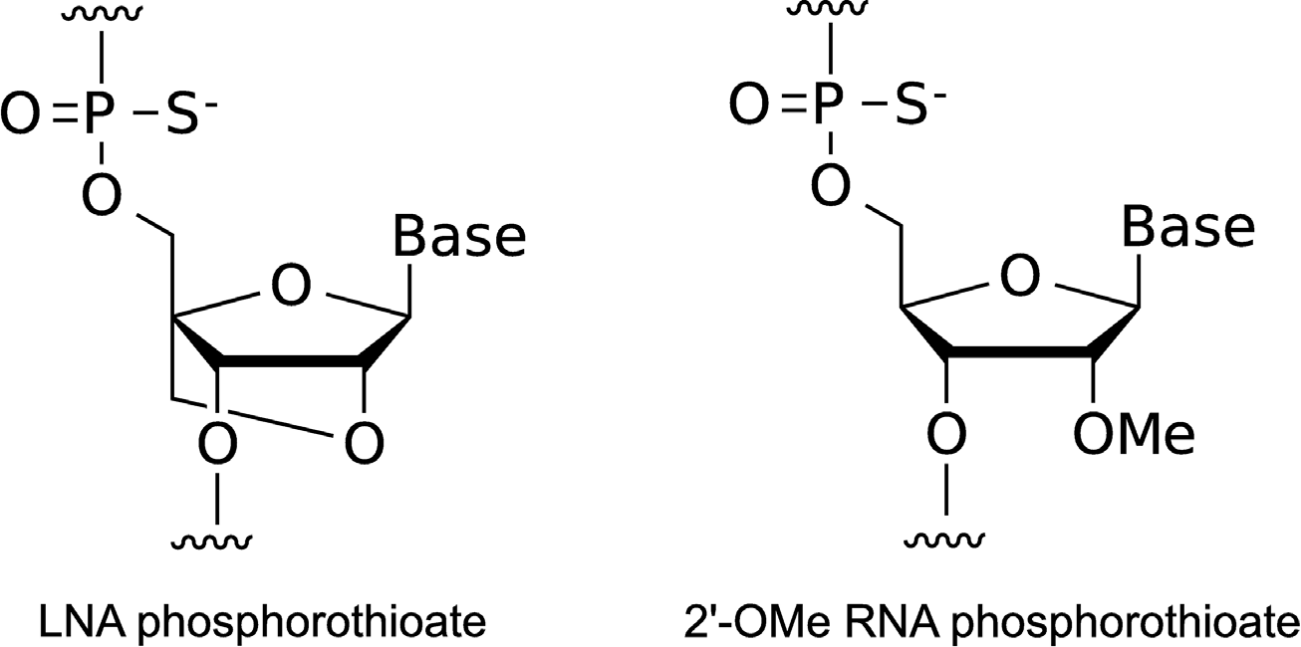
Structures of the building blocks for SSOs. PS LNA and PS 2’-OMe RNA.

All SSOs used in this study are shown in Supplementary Tables S1–S9. Two types of modification, LNA and 2’-OMe, were incorporated into the SSO sequences, in which the phosphodiester linkages were completely replaced by PS linkages (Figure 1). All SSOs were designed to have sequences complementary to human dystrophin gene and were synthesized and purified by Gene Design Inc. (Osaka, Japan).

### Plasmid construction

The reporter construct was generated using standard cloning techniques published in a previous study (32). A FLAG (DYKDDDDK)-coding oligonucleotide was constructed by annealing the forward oligonucleotide 5' -AGCTTACCATGGATTACAAGGACGACGACGACAAGGGGGTAC-3' (including HindIII and KpnI sites, underlined) and reverse oligonucleotide 5' -CCCCTTGTCGTCGTCGTCCTTGTAATCCATGGTA-3' . The annealed oligonucleotide was cloned into the HindIII-KpnI sites of the pcDNA5/FRT vector (Invitrogen, Carlsbad, CA, USA) (termed pcDNA5/FRT-FLAG). The EGFP fragment was obtained by PCR using the forward primer 5' -CCCGGGTGTGAGCAAGGGCGAGGAGCTGT-3' (including a SmaI site, underlined) and reverse primer 5' -ATAGGGCCCTTACTTGTACAGCTCGTCCAT-3' (including an ApaI site, underlined). The obtained EGFP fragment was cloned into the EcoRV-ApaI sites of pcDNA5/FRT-FLAG (termed pcDNA5/FRT-FLAG-EGFP). The DsRed fragment was obtained by PCR from the pDsRed-Express-N1 vector (Clontech, Mountain View, CA, USA) using the forward primer 5' -ATATGGATCCAACCGGTGTGGCCTCCTCCGAGGACGTCA-3' (including BamHI and AgeI sites, underlined) and reverse primer 5' -CGGTCTACAGGAACAGGTGGTGGC-3' . The obtained DsRed fragment was cloned into the BamHI-SmaI sites of the pcDNA5/FRT-FLAG-EGFP vector (termed pcDNA5/FRT-FLAG-DsRed-EGFP). A nuclear localization signal (NLS) was constructed by annealing the forward oligonucleotide 5' -ATGCCCCAAAAAAAAAACGCAAAGTGGAGGACCCAAAGGTACCAAAG-3' (including a KpnI site, underlined) and reverse oligonucleotide 5' -GATCCTTTGGTACCTTTGGGTCCTCCACTTTGCGTTTTTTTTTTGGGGCATGTAC-3' . The annealing oligonucleotide was cloned into the KpnI-BamHI sites of pcDNA5/FRT-FLAG-DsRed-EGFP (termed pcDNA5/FRT-FLAG-NLS-DsRed-EGFP).

A human dystrophin minigene containing exons 57–59 was isolated as follows. Because the intron 57 sequence consists of 17 684 bp and is thus too long to insert into a plasmid, we designed a human dystrophin minigene by removing the sequence of intron 57 from position +207 to +17 486. Thus, exon 57, together with a short flanking intronic sequence, was obtained by PCR from the HepG2 genome using the forward primer 5' -AACGGTACCAACGCTGCTGTTCTTTTTCA-3' (including a KpnI site, underlined) and reverse primer 5' -GTGTTTGTAATGGACGATTTCTTAAAGGGTATT-3' . Another fragment containing a short 3' sequence of intron 57 to exon 59 was also obtained by PCR using the forward primer 5' -AAATCGTCCATTACAAACACAGCGCTTTCC-3' and reverse primer 5' -AGACCGGTACTCCTCAGCCTGCTTTCGTA-3' (including an AgeI site, underlined). These two fragments were mixed, and a second round of PCR was performed. Finally, after the second round of PCR, the newly synthesized full-length PCR product was cloned into the KpnI-AgeI sites of the pcDNA5/FRT-FLAG-NLS-DsRed-EGFP vector to generate a dystrophin reporter minigene (termed pcDNA5/FRT-FLAG-NLS-DMD-Exon57_58_59(short-Intron57)-DsRed-EGFP). All constructs were verified by sequencing.

### Generation of a stable cell line

Flp-In 293 cells (Invitrogen) were cultured in Dulbecco's modified Eagle Medium (DMEM) (Nacalai Tesque, Kyoto, Japan) containing 10% fetal bovine serum (FBS) (Biowest, Nuaillé, France), 100 units/ml penicillin and 100 μg/ml streptomycin (Nacalai Tesque) and maintained in a 5% CO_2_ incubator at 37°C. Flp-In 293 cells were co-transfected with pcDNA5/FRT-FLAG-NLS-DMD-Exon57_58_59(short-Intron57)-DsRed-EGFP and pOG44 (the flp recombinase expression plasmid) (Invitrogen). Stable cell lines were selected by 50 μg/ml hygromycin B (Invitrogen).

### SSOs transfection

Stable cell lines were seeded one day before transfection at a density of 8.0 × 10^4^ cells/well on 24-well plates. At 30%–40% confluence, SSOs were transfected into cells by using Lipofectamine 2000 (Invitrogen) according to the manufacturer's instructions. After 24 h, the cells were harvested.

### RNA isolation and cDNA synthesis

Total RNA samples were isolated from the cells using the QuickGene 800 and QuickGene RNA cultured cell kit S (KURABO, Osaka, Japan) according to the manufacturer's instructions. First-strand cDNA was synthesized from 150 ng of the total RNA of each cell sample using the ReverTra Ace qPCR RT Master Mix (TOYOBO, Osaka, Japan) according to the manufacturer's instructions.

### Primary myoblast cell culture, SSO transfection and RNA isolation

Primary human skeletal muscle myoblasts (HSMM) derived from healthy Caucasian donor (female aged 17 years) were purchased from Lonza (Walkersville, MD, USA). HSMM cells were cultured in SkBM-2 basal medium (Lonza) supplemented with 10% FBS, epidermal growth factor (EGF), dexamethasone, L-glutamine, gentamycin sulfate and amphotericin B (SingleQuots, Lonza) and maintained in a 5% CO_2_ incubator at 37ºC. For SSO transfection, cells were seeded 2 days before transfection at a density of 1.0 × 10^5^ cells/well on 24-well collagen type I coated plates. After 24 h, cells were differentiated by changing the growth medium to differentiation medium (DMEM/F-12 (Life Technologies, Carlsbad, CA, USA) containing 2% horse serum (Life Technologies) and antibiotic-antimycotic solution (100 units/ml penicillin, 100 μg/ml streptomycin, 0.25 μg/ml amphotericin B) (Life Technologies)) for 24 h. Cells were transfected with 500 nM SSOs using Lipofectamine 2000 according to the manufacturer's instructions. Twenty-four hours after transfection, total RNA samples were isolated from the cells using the QuickGene 800 and QuickGene RNA cultured cell kit S according to the manufacturer's instructions. First-strand cDNA was synthesized from 50 ng of the total RNA of each cell sample using the ReverTra Ace qPCR RT Master Mix according to the manufacturer's instructions.

### RT-PCR analysis

The cDNA was used as a template for individual PCR reactions using specific primer sets (Supplementary Table S10), which were designed using the Primer3 program written by the Whitehead Institute (33). PCR reactions were conducted using KOD FX Neo DNA polymerase (TOYOBO), and the PCR products were analyzed on a 2% agarose gel stained with ethidium bromide, with specific bands purified for sequence analysis. The intensity of each band was quantified by using ImageJ software (National Institutes of Health; freeware from http://rsb.info.nih.gov/ij/) and normalized according to the nucleotide composition. The exon skipping percentage was calculated as the amount of exon 58-skipped transcript relative to the total amount of the exon 58-skipped and full-length transcripts (34). Glyceraldehyde-3-phosphate dehydrogenase (GAPDH) was used as an internal control.

### Quantitative real-time RT-PCR analysis

The cDNA was used as a template for individual PCR reactions using exon skipping specific primer sets (Supplementary Table S11), which were designed using the Primer Express program (Applied Biosystems, Foster City, CA, USA) and Primer3 program. PCR reactions were conducted using SYBRGreen Real-time PCR Master Mix (TOYOBO) according to the manufacturer's instructions, except that the annealing time was reduced to 15 s. The quantitative PCR analysis was performed using the StepOnePlus device (Applied Biosystems). Amplification specificity was verified by visualizing the PCR products on an ethidium bromide-stained 2% agarose gel. GAPDH was used to normalize the expression data.

### Ultraviolet (UV) melting experiment

UV melting experiments were conducted using a Shimadzu UV-1650PC UV-Vis spectrophotometer equipped with a *T*_m_ analysis accessory TMSPC-8 (Shimadzu, Kyoto, Japan). Equimolecular amounts of SSO and complementary RNA oligonucleotide were dissolved in 10 mM sodium phosphate buffer (pH 7.2) containing 10 mM NaCl to give a final strand concentration of 2.0 μM. The samples were boiled for 3 min, followed by slow cooling to room temperature. The absorption was recorded at 260 nm in the forward and reverse direction from 5°C to 95°C at a scan rate of 0.5°C/min. The first derivative was calculated from the smoothed UV melting profile. The peak temperatures in the derivative curve were designated as the melting temperature, *T*_m_.

### *In silico* analysis to search for target sequence

To know the number of genes that contain the sequence perfectly matched to the target sequence of AONs, we used GGRNA, a Google-like fast search engine for genes and transcripts (http://GGRNA.dbcls.jp/) (35). In this analysis, we considered splicing variants with the same gene ID as one gene and excluded the genes which do not encode proteins.

## RESULTS

### Screening for LNA SSOs effective for inducing exon skipping

We performed a screening analysis to obtain effective LNA SSOs that induced skipping of exon 58 of the human dystrophin gene. Prior to starting the screening of the SSOs, we developed a minigene reporter plasmid containing exons 57–59 of the human dystrophin gene. Subsequently, we established a stable reporter cell line in which the reporter plasmid was incorporated into the genomic DNA and used as a splicing assay system. To evaluate the efficacy of the designed SSOs, the reporter cells were transfected with each SSO, and exon skipping was analyzed by RT-PCR (Supplementary Figure S1).

**Figure 2.**
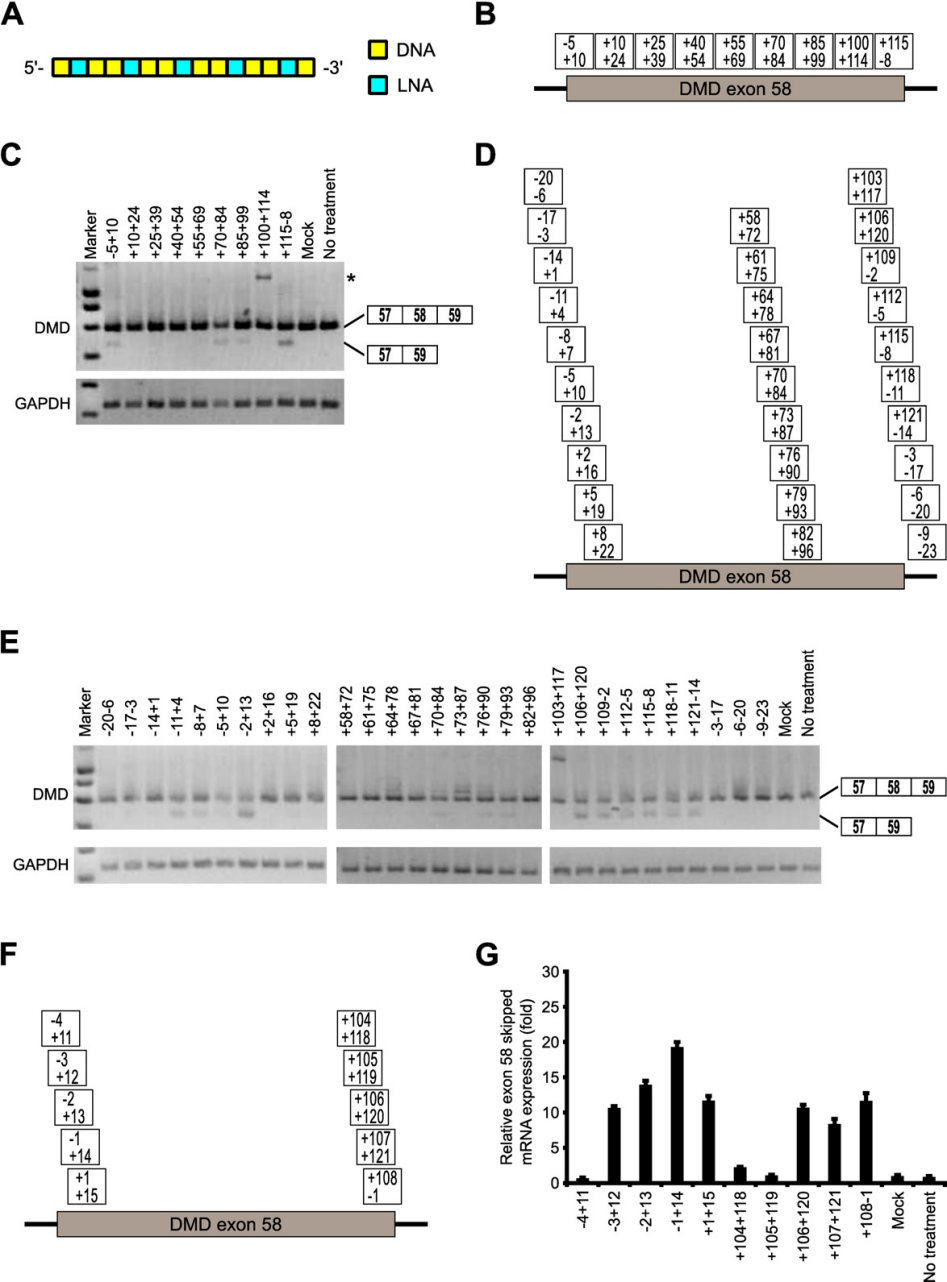
Screening of 15-mer LNA/DNA mixmer SSOs designed to induce dystrophin exon 58 skipping. (**A**) Schematic representation of the position of LNA in the 15-mer SSO used in this screening. Each box represents one nucleotide; the blue box and yellow box indicate LNA and DNA, respectively. (**B, D, F**) Annealing sites of SSOs targeted to dystrophin exon 58 are indicated by the boxes for the first (B), second (D) and third screening (F). (**C, E**) The reporter cells were transfected with the indicated LNA/DNA mixmer SSOs (100 nM) for 24 h. RT-PCR analyses showing the full-length upper band (587 bp) and the skipped lower band (466 bp). The band marked by an asterisk represents an intron 58 inclusion product. GAPDH was used as an internal control. (C) and (E) express the results of the first and second screening, respectively. (**G**) The levels of exon 58-skipped mRNA fragments were measured by quantitative real-time RT-PCR and normalized against the signal of GAPDH mRNA, relative to the value in the mock set as 1. Values represent the mean ± standard deviation of triplicate samples. Reproducible results were obtained from two independent experiments. Mock: treated with Lipofectamine only; no treatment: no transfection.

In this screening study, we designed a series of 15-mer LNA/DNA mixmers with a LNA substitution at every third nucleotide position. These mixmers contained five LNA units in the SSO sequence, in which the phosphodiester linkages were completely replaced by PS linkages (Figure 1). To prevent RNase H-dependent RNA degradation, we designed the number of continuous natural nucleotides in the SSO to be less than two (Figure 2A) (36). The screening was composed of three steps. At the first step, nine non-overlapping LNA SSOs were designed to tile across the entire target exon 58 sequence to detect a prospective target site (Figure 2B and Supplementary Table S1). Reporter cells were transfected with 100 nM SSOs for 24 h. Total RNA samples were prepared, and RT-PCR analyses showed that three LNA SSOs, i.e. -5+10, +70+84 and +115-8, were effective in slightly inducing exon skipping of exon 58 (the rate of exon skipping was 10%–20%) (Figure 2C and Supplementary Figure S2A).

In the second step, to detect the more active SSOs, we synthesized three sets of 15-mer LNA SSOs shifted by three bases around each expected target sequence, i.e. -5+10, +70+84 and +115-8 (Figure 2D and Supplementary Table S2). These SSOs (100 nM) were transfected into the reporter cell line, and total RNA samples were prepared after a further 24 h incubation. The RT-PCR results suggested that both the 5' and 3' splice sites in addition to the 27-base region from +70 to +96 of exon 58 are hot spots for inducing exon skipping (Figure 2E and Supplementary Figure S2B). In addition, this region was predicted as an exonic splicing enhance (ESE) site by ESEfinder3.0 (Supplementary Figure S3) (37,38). In this case, we decided to select two SSOs, -2+13 and +106+120, as templates for the next screening step given their high ability to modulate splicing (the rate of exon skipping increased to ca. 50%) (Supplementary Figure S2B).

Finally, we designed a further four LNA SSOs shifted by one nucleotide around each SSO, i.e. -2+13 and +106+120 (Figure 2F and Supplementary Table S3). In the third step, we assessed the expression of exon 58-skipped mRNA by means of quantitative real-time RT-PCR to rigorously evaluate the abilities of the SSOs. Both the SSO (-1+14) in the 5' splice site and the SSO (+108-1) in the 3' splice site showed higher exon 58 skipping activity (Figure 2G). Surprisingly, in some cases SSOs that are frameshifted by one nucleotide resulted in loss of SSO activity (e.g. -4+11 versus -3+12). These findings demonstrate that exon 58 skipping can be modulated by 15-mer LNA/DNA mixmer SSOs targeting near the 5' and 3' -splice sites of exon 58, and that this activity is strongly dependent on the target sequence.

### Evaluation of the effect of number of LNAs and *T*_m_ value on splicing

**Figure 3.**
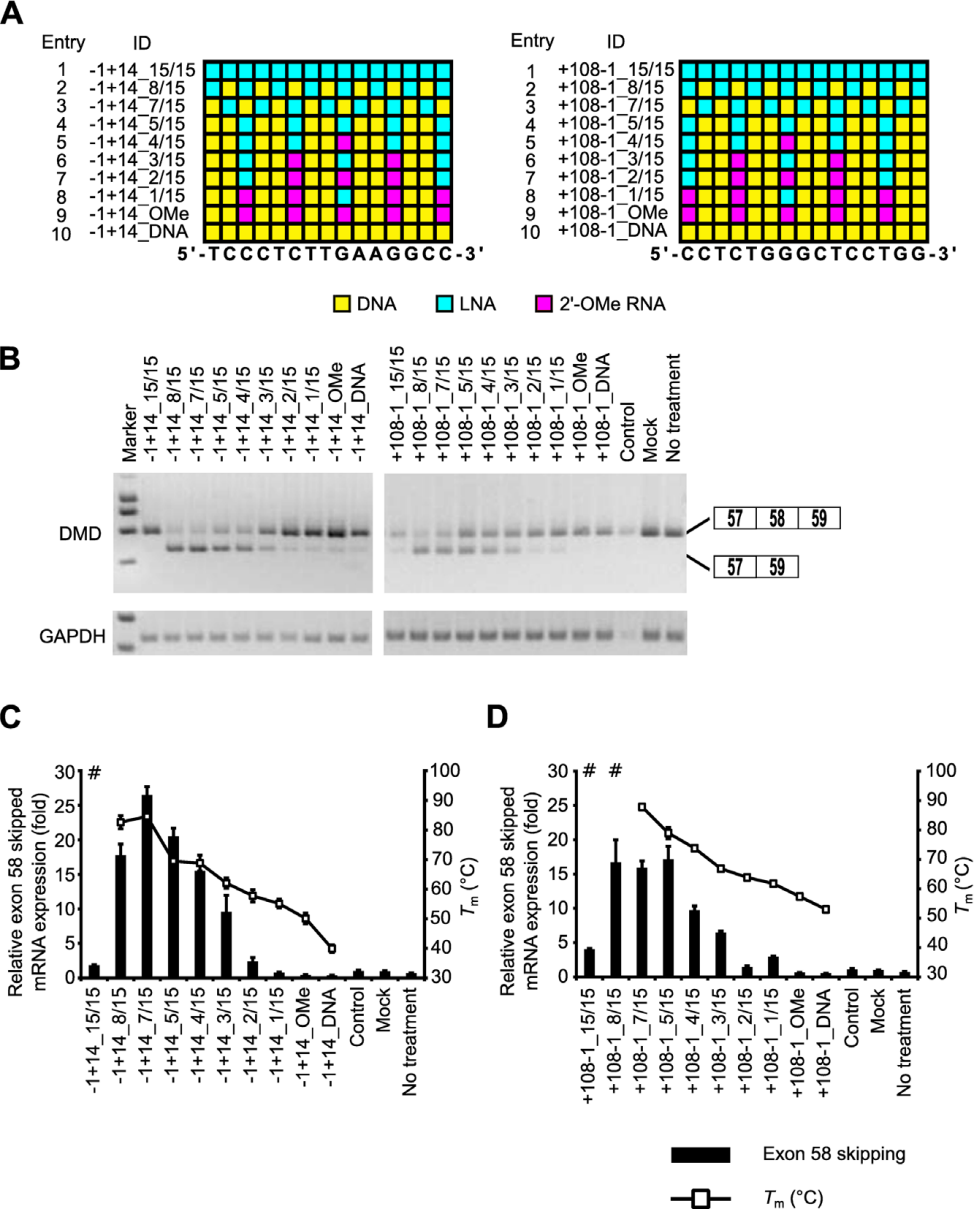
Evaluation of exon skipping activity of 15-mer SSOs with various numbers of LNAs and *T*_m_ values. (**A**) Schematic representation of the position of LNA in the 15-mer SSOs used in this study. Each box represents one nucleotide; the blue box, red box and yellow box indicate LNA, 2' -OMe RNA and DNA, respectively. (**B**) The reporter cells were transfected with the indicated LNA/DNA mixmer SSOs (30 nM) for 24 h. RT-PCR analyses were performed as described in Figure 2C. LNA SSO (+10+24), which showed no exon skipping effects, was used as a control. (**C** and **D**) The levels of exon 58-skipped mRNA fragments were measured by quantitative real-time RT-PCR (for details see Materials and Methods and Figure 2G). Values represent the mean ± standard deviation of triplicate samples. Reproducible results were obtained from two independent experiments. The *T*_m_ of each SSO with a complementary RNA under low-sodium conditions is also shown. # indicates that no sigmoidal melting curve was observed, even at higher *T*_m_ values. The data are the mean ± standard deviation (*n* = 4). (C) and (D) express exon skipping results of using SSOs targeted the 5' and 3' splice sites, respectively.

In the following experiments, we selected two sequences identified from the above screening for exon skipping (-1+14 and +108-1). To investigate the relationship between the number of LNAs in the sequence of the SSOs and skipping activity, we synthesized a series of 15-mer SSOs that had various numbers of LNAs (Figure 3A). To protect the SSOs against nuclease degradation and prevent RNase H-induced pre-mRNA digestion, 2' -OMe RNAs were introduced into SSOs if fewer than five LNAs were in the sequence. We determined the *T*_m_ values of these SSOs with complementary RNA by UV melting experiments performed under low-sodium conditions (10 mM phosphate buffer (pH 7.2) containing 10 mM NaCl) (Supplementary Tables S4 and S5). The lower ionic concentration of the solvent tended to decrease *T*_m_ relative to the typical ionic concentrations, such as 100 mM NaCl (Supplementary Table S4). LNA SSO (+10+24) did not show an exon skipping effect (Figure 2C) and was thus used as a control.

We transfected reporter cells with 30 nM SSOs, and the cells were then incubated for 24 h. Then, total RNA samples were prepared, and exon 58-skipped mRNA levels were determined by both RT-PCR and quantitative real-time RT-PCR. RT-PCR analysis indicated that increasing the number of LNAs enhanced exon skipping activity (the rate of exon skipping reached 80%) (Figure 3B and Supplementary Figure S4A and B). Similar results were obtained by quantitative real-time RT-PCR assays, and SSOs containing between five and eight LNAs induced exon skipping at high levels (Figure 3C and D). On the other hand, SSOs fully modified with LNA showed very low activity, and their *T*_m_ values were higher than 95ºC. In our experiments, efficient exon skipping activity was obtained when LNA/DNA mixmer SSOs were designed with a *T*_m_ in the range of 60°C–90°C (low sodium conditions). In comparison to the LNA SSOs, both the 2' -OMe SSO and DNA SSO hardly affected exon skipping. These results indicate that the number of LNA in the SSO sequence and the *T*_m_ of the SSOs play important roles in exon skipping.

### Influence of SSO length on exon skipping

**Figure 4.**
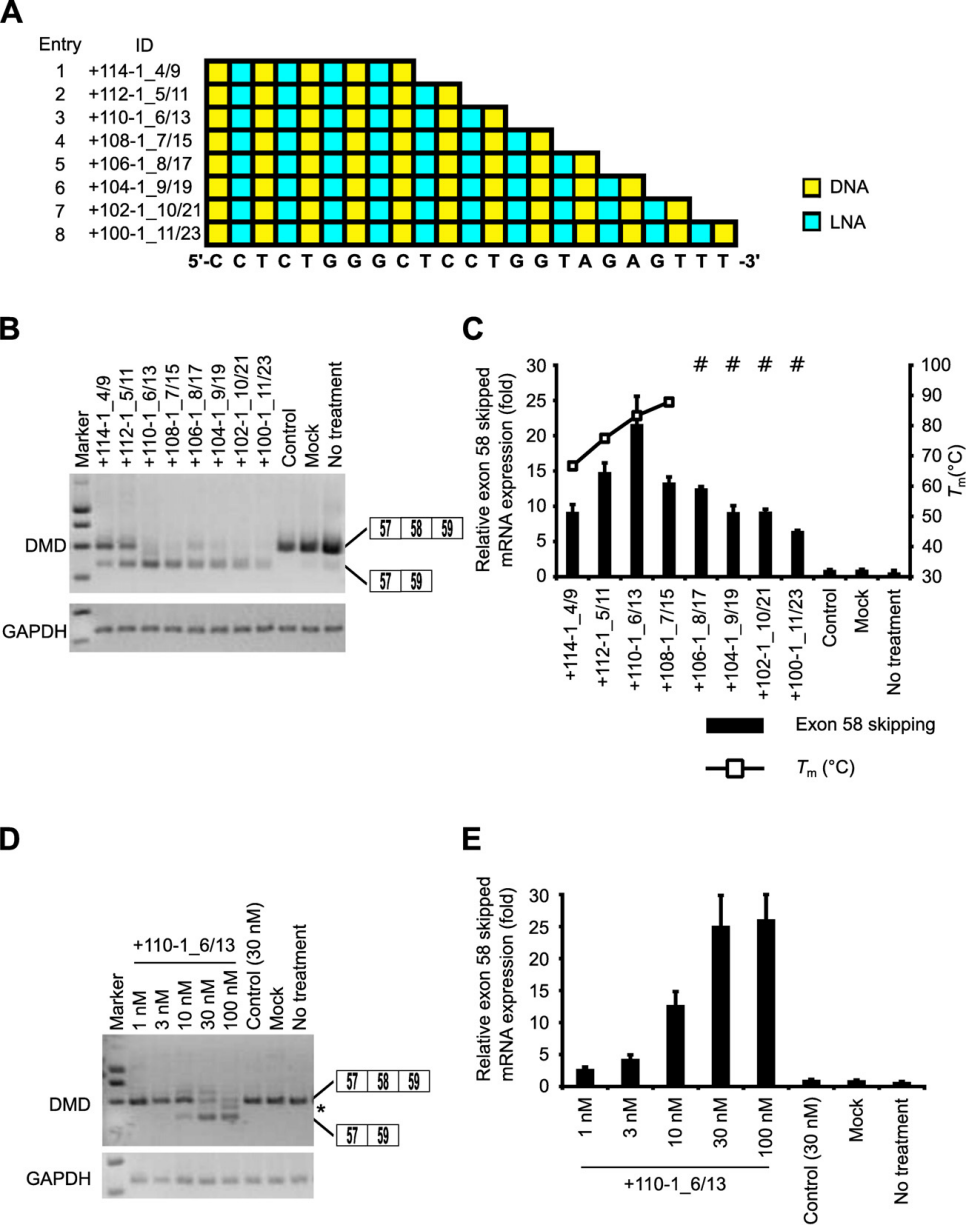
Comparison of the exon skipping activity of various lengths of LNA/DNA mixmer SSOs. (**A**) Schematic representation of the position of LNA in the SSOs used in this study. (**B** and **D**) The reporter cells were transfected with the indicated LNA/DNA mixmer SSOs (30 nM (B) or various concentrations (1–100 nM) (D)) for 24 h. RT-PCR analyses were performed as described in Figure 3B. The band marked by an asterisk represents a partial intron 58 inclusion product. (**C** and **E**) The levels of exon 58-skipped mRNA fragments were measured by quantitative real-time RT-PCR (for details see Materials and Methods and Figure 2G). Values represent the mean ± standard deviation of triplicate samples. Reproducible results were obtained from two independent experiments. The *T*_m_ of each SSO with a complementary RNA under low-sodium conditions is determined as described in Figure 3C. The data are the mean ± standard deviation (*n* = 4).

To determine whether the SSO length affects splicing modulation, we tested SSOs targeting the 3' splice site. The length of the SSOs ranged from 9 to 23 nucleotides. Taking into account the *T*_m_ values of the short SSOs, we designed eight SSOs that contained 50% LNAs in their sequences (Figure 4A and Supplementary Table S6). We also determined dissociation constant (*K*_d_) for LNA/DNA mixmer SSOs to mRNA (Supplementary Materials and Methods and Supplementary Table S6).

Reporter cells were transfected with 30 nM SSOs. Total RNA samples were prepared after a further 24 h incubation, and we assessed the expression of exon 58-skipped mRNA by means of RT-PCR and quantitative real-time RT-PCR. RT-PCR revealed that the longer LNA SSOs induced exon skipping with high efficiency (the rate of exon skipping was ∼75%) (Figure 4B and Supplementary Figure S5). Intriguingly, when the levels of exon 58-skipped mRNA were analyzed by quantitative real-time RT-PCR, the 13-mer SSO (+110-1_6/13) produced high amounts of exon 58 skipping mRNAs in a concentration-dependent manner (Figure 4C–E and Supplementary Figure S6A). A concentration-dependent increase was also observed for other SSO (-1+14_7/15) targeting the 5' splice site (Supplementary Figure S6B–D). Amazingly, the 9-mer LNA SSO (+114-1_4/9) (*T*_m_ = 66.7ºC) induced exon skipping, in a similar manner as 19-, 21- and 23-mer SSOs (Figure 4C).

### Characterization of 9-mer LNA SSOs

**Figure 5.**
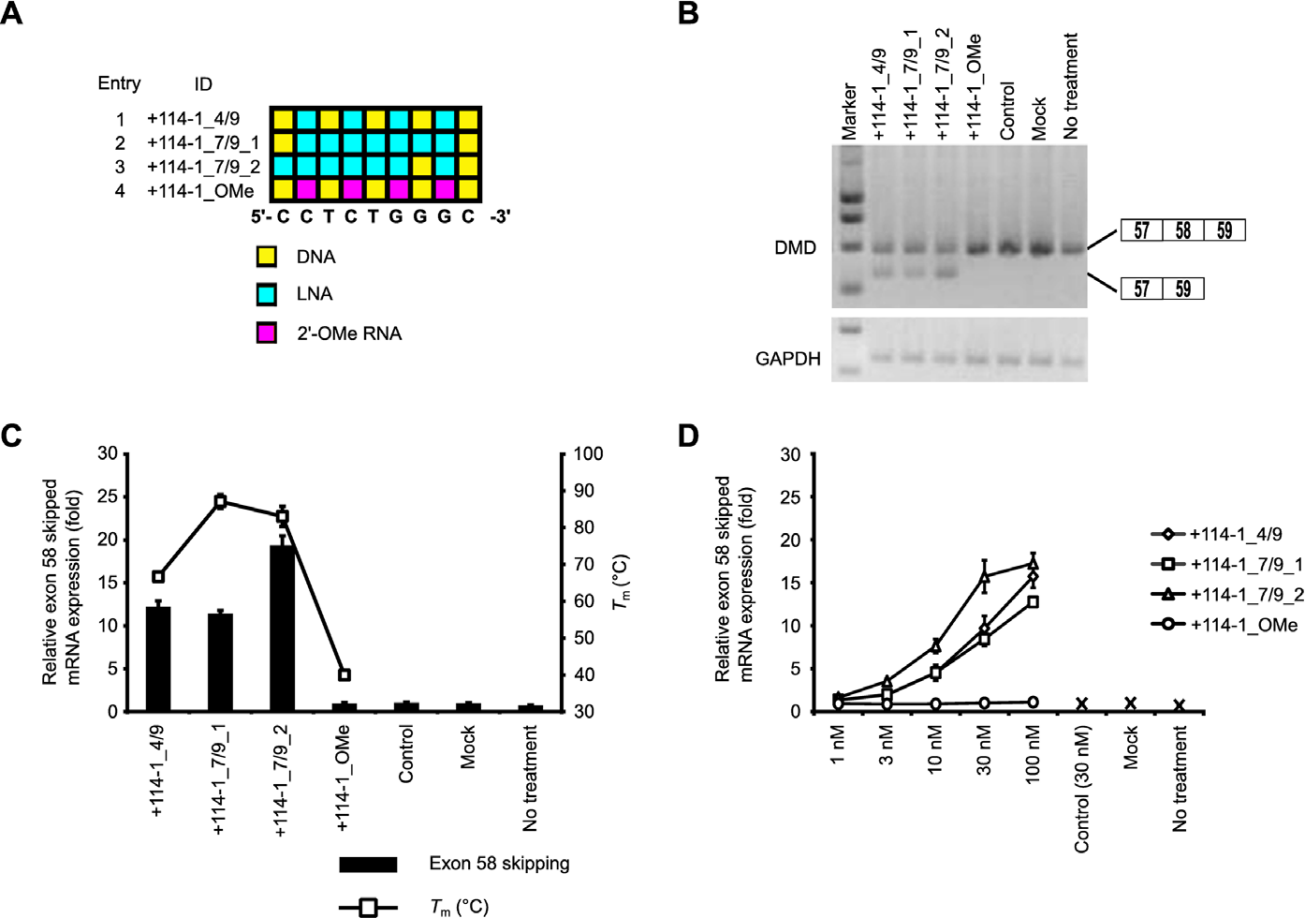
Exon skipping activity of 9-mer LNA/DNA mixmer SSOs. (**A**) Schematic representation of the position of LNA in the 9-mer SSOs used in this study. (**B**) The reporter cells were transfected with the indicated LNA/DNA mixmer SSOs (30 nM) for 24 h. RT-PCR analyses were performed as described in Figure 3B. (**C** and **D**) Quantitative real-time RT-PCR analyses of RNA samples from reporter cells treated with SSOs at 30 nM (C) or at various concentrations (1–100 nM) (D) for 24 h were performed as described in Figure 2G. Values represent the mean ± standard deviation of triplicate samples. Reproducible results were obtained from two independent experiments. The *T*_m_ of each SSO with a complementary RNA under low-sodium conditions is also shown. The data are the mean ± standard deviation (*n* = 4).

Although no reports to date have indicated that 9-mer SSOs induce exon skipping, we found that even a 9-mer SSO (+114-1_4/9) (four LNA and five DNA) could modulate splicing, albeit weakly. Therefore, to further improve the efficiency of exon skipping, we designed another two 9-mer SSOs containing seven LNAs having the same sequence as +114-1_4/9 (Figure 5A and Supplementary Table S7). As expected, the *T*_m_ values of both SSOs (+114-1_7/9_1 and +114-1_7/9_2) were higher than that of SSO (+114-1_4/9) (87.1ºC, 83.1ºC, and 66.7ºC, respectively) (Supplementary Table S7).

The reporter cells were treated with 30 nM SSOs for 24 h and then subsequently lysed with the total RNA extracted. Interestingly, 9-mer LNA SSO (+114-1_7/9_2) presented 1.5-fold higher activity than the other 9-mer LNA SSOs (Figure 5B and C and Supplementary Figure S7). It seems that the position of LNA analogues in the SSO sequence may be a key factor for exon skipping. Moreover, this 9-mer SSO induced exon skipping in a concentration-dependent manner (Figure 5D and Supplementary Figure S8A–E). In contrast, the 9-mer 2' -OMe SSO (*T*_m_ = 40.0ºC) exhibited no exon skipping activity at all. In this study, we report for the first time that 9-mer LNA SSOs have sufficient activity to induce exon skipping.

### Short 7-mer LNA SSO-induced exon skipping in the reporter cells

**Figure 6.**
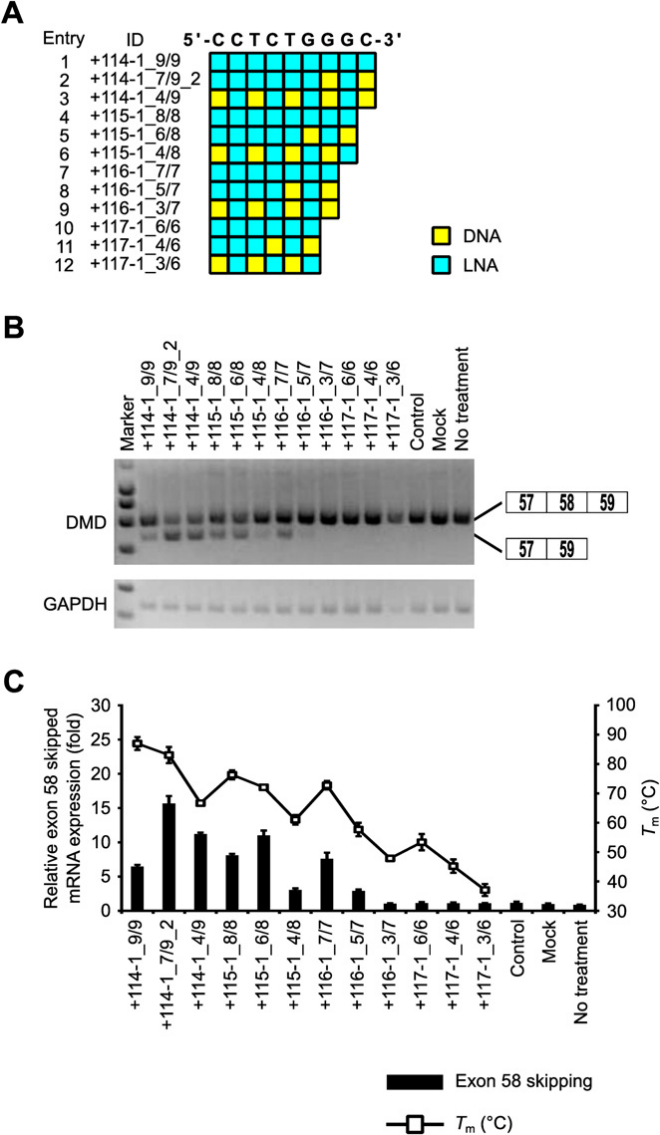
Comparison of exon skipping activity of short (6- to 9-mer) LNA SSOs. (**A**) Schematic representation of the position of LNA in the SSOs used in this study. (**B**) The reporter cells were transfected with the indicated LNA/DNA mixmer SSOs (30 nM) for 24 h. RT-PCR analyses were performed as described in Figure 3B. (**C**) The levels of exon 58-skipped mRNA fragments were measured by quantitative real-time RT-PCR (for details see Materials and Methods and Figure 2G). Values represent the mean ± standard deviation of triplicate samples. Reproducible results were obtained from two independent experiments. The *T*_m_ of each SSO with a complementary RNA under low-sodium conditions is also shown. The data are the mean ± standard deviation (*n* = 3–4).

To determine the minimum length of LNA SSO required for inducing exon skipping, we tested short LNA SSOs (6- to 9-mer) containing various numbers of LNAs (Supplementary Table S8). We designed three sets of LNA SSOs (fully modified LNA SSOs which have as high *T*_m_ value as possible, LNA/DNA mixmer SSOs containing 50% LNAs, such as Figure 4A, and LNA/DNA mixmer SSOs containing two DNAs like a +114-1_7/9_2, respectively) for each length (Figure 6A).

Reporter cells were transfected with 30 nM SSOs. Total RNA samples were prepared after a further 24 h incubation, and we assessed the expression of exon 58-skipped mRNA by means of RT-PCR and quantitative real-time RT-PCR. RT-PCR revealed that 9-, 8- and 7-mer LNA SSOs induced exon skipping, while 6-mer LNA SSOs did not show any activity (Figure 6B and Supplementary Figure S9). In the case of short LNA SSOs, the longer SSOs tend to have higher activity and exon skipping activity was obtained when LNA SSOs were designed with a *T*_m_ higher than 60°C (low sodium conditions) (Figure 6C). These results indicate that even very short 7-mer LNA SSO provides exon skipping activity.

### Effect of mismatches on exon skipping activity and sequence specificity

**Figure 7.**
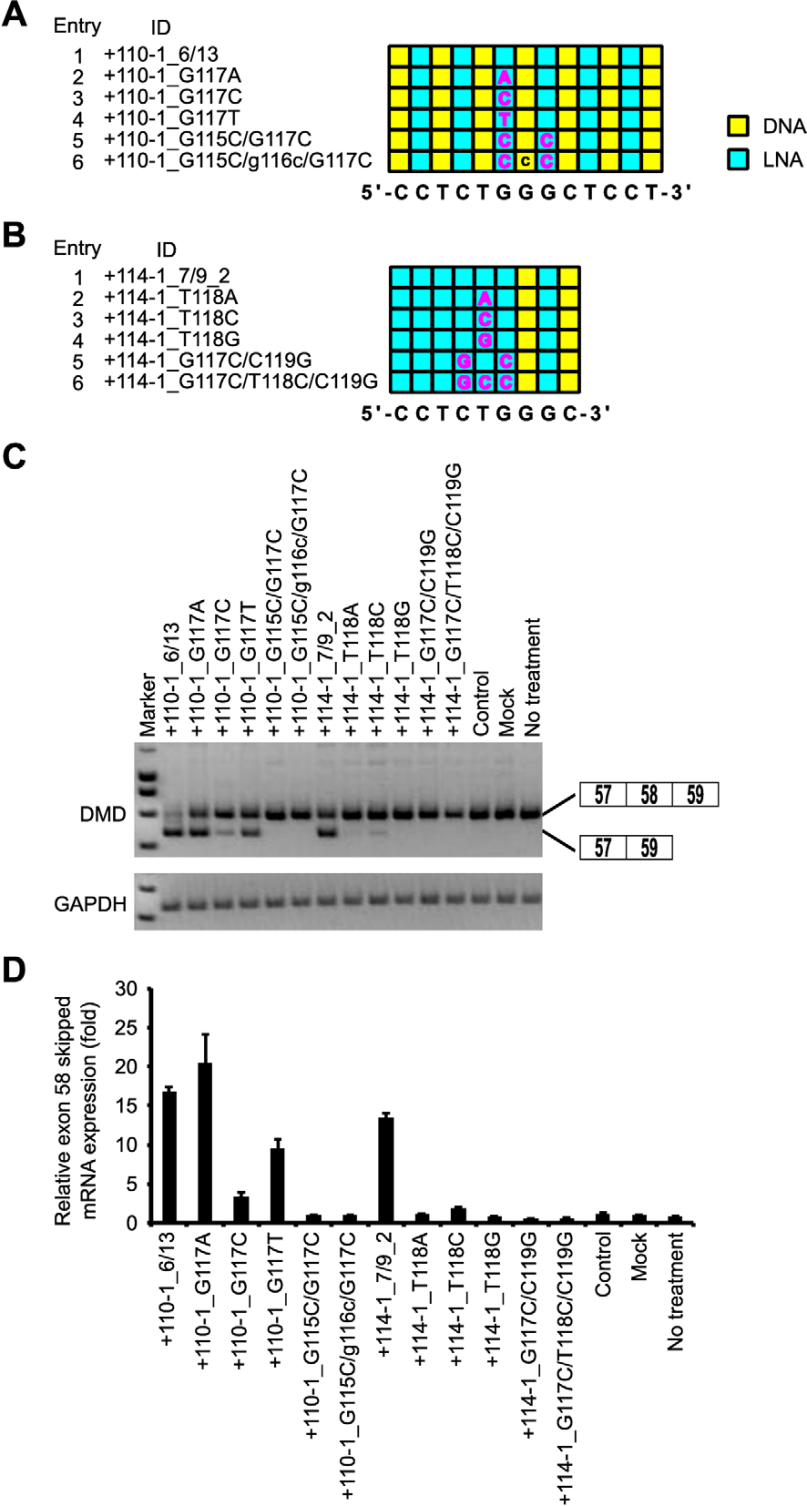
Assessment of specificity of LNA SSO. (**A** and **B**) Schematic representation of the position of LNA in the 13-mer SSOs (A) and the 9-mer SSOs (B) used in this study. The sequence in the box indicates a mismatch. Capital letter A, G, T: LNA; C: 5-methyl cytosine LNA; lowercase letter: DNA. (**C**) The reporter cells were transfected with the indicated LNA/DNA mixmer SSOs (30 nM) for 24 h. RT-PCR analyses were performed as described in Figure 3B. (**D**) The levels of exon 58-skipped mRNA fragments were measured by quantitative real-time RT-PCR (for details see Materials and Methods and Figure 2G). Values represent the mean ± standard deviation of triplicate samples. Reproducible results were obtained from two independent experiments.

To assess the specificity of LNA SSOs, we introduced one, two or three mismatches in both 13- and 9-mer LNA SSOs (Figure 7A and B and Supplementary Table S9). The reporter cells were transfected with 30 nM LNA SSOs. Twenty-four hours after transfection, total RNAs were extracted and the expression levels of exon 58-skipped mRNA were analyzed by both RT-PCR and quantitative real-time RT-PCR (Figure 7C and D and Supplementary Figure S10). We observed that when one LNA mismatch is introduced into 13-mer LNA SSO (six LNA and seven DNA) (+110-1_G117A, +110-1_G117C and +110-1_G117T), they can still induce exon skipping of exon 58. In contrast, mismatched LNA SSOs with two or three mismatches (+110-1_G115C/G117C and +110-1_G115C/g116c/G117C) did not show exon skipping activity. In the case of 9-mer LNA SSO (seven LNA and two DNA), one to three LNA mismatches abrogated the effect on exon 58 skipping. These results indicate that a 9-mer LNA SSO shows a better mismatch discrimination than a 13-mer LNA SSO. We next searched for target sequence of both 13- and 9-mer LNA SSOs using GGRNA (35). It is revealed that there were 914 genes that have perfect match with the 9-mer LNA SSO (+114-1_7/9_2). On the other hand, only 8 genes contain sequences perfectly matched to the 13-mer LNA SSO (+110-1_6/13) (Table 1). Thus, although 9-mer LNA SSOs improve mismatch discrimination in comparison with 13-mer LNA SSOs, 9-mer SSOs may be too short to target unique sites.

**Table 1.** A number of genes that contain the sequence complementary to each AON. Sequences are shown from 5' to 3'.

ID	Target sequence	Length (bp)	No. of genes containing the target sequence
+114-1_7/9_2	GCCCAGAGG	9	914
+110-1_6/13	AGGAGCCCAGAGG	13	8

### Induction of exon 58 skipping of endogenous human dystrophin transcript by using LNA SSO

**Figure 8.**
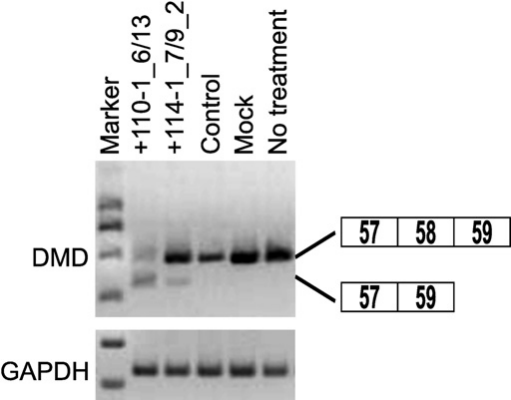
Exon skipping by LNA SSOs in primary human skeletal muscle cells. HSMM cells were transfected with the indicated LNA/DNA mixmer SSOs (500 nM) for 24 h. RT-PCR analyses show the full-length upper band (575 bp) and the skipped lower band (454 bp). LNA SSO (+10+24), which showed no exon skipping effects, was used as a control. GAPDH was used as an internal control.

Finally, to examine whether LNA SSOs modulate the splicing of endogenous human dystrophin transcript, we used primary HSMM cells. Cells were treated with the differentiation medium 24 h prior to transfection. Then, HSMM cells were transfected with 500 nM SSOs. Total RNA samples were prepared after a further 24 h incubation, and we assessed the expression of exon 58-skipped endogenous human dystrophin mRNA by means of RT-PCR. Although the 9-mer LNA SSO (+114-1_7/9_2) induced weak exon skipping, the 13-mer LNA SSO (+110-1_6/13) induced a high amount of exon skipping (Figure 8). In contrast, control SSO did not affected exon skipping. These data indicate that LNA SSOs are able to induce exon skipping of endogenous human dystrophin in cultured muscle cells.

## DISCUSSION

In this study, we designed and evaluated the exon skipping ability of a series of LNA SSOs complementary to the human dystrophin exon 58 sequence. We also indicated that LNA SSOs induce endogenous dystrophin exon 58 skipping in primary human skeletal muscle cells.

To develop the splicing assay system we used native human dystrophin sequences because it is thought that the RNA structures play an important role in the regulation of splicing (39). According to previous reports, exon skipping target to exon 58 is applicable to only 0.1% of DMD patients (40). The average length of human dystrophin introns tends to be higher than 25 000 bp; therefore, the entire gene is inappropriate to introduce into a plasmid. However, intron 58, whose length is only 608 bp, has a much shorter sequence than other introns (41). We therefore selected exon 58 to construct our assay system despite the rarity of patients with mutations correctable by exon 58 skipping. On the other hand, the intron 57 sequence, consisting of 17 684 bp, is too long to insert into a reporter plasmid. Thus, although splicing regulatory sequences are located not only near exon–intron junctions but also intronic regions, we decided to use the human dystrophin minigene encompassing exons 57–59 by removing the sequence of intron 57 from position +207 to +17 486. Moreover, we established a stable Flp-In 293 cell line in which the reporter plasmid is incorporated into the genomic DNA, and that this screening cell line is easier to maintain than primary cells due to the robust growth. Because LNA SSOs selected by using this screeing cell line induced exon 58 skipping of endogenous dystrophin gene in the HSMM (Figure 8), this cell line is a useful evaluation tool for screening a lot of SSOs, as used in this study.

First, to identify effective SSOs capable of modulating exon skipping, 43 LNA SSOs, each with a length of 15 bases, were evaluated by RT-PCR. Exon skipping was induced at high levels by targeting either acceptor or donor sites with SSOs (Figure 2). In addition, this systematic screen also identified the 27-bp region from +70 to +96 of exon 58 as an appropriate target for SSOs. Interestingly, this region was predicted to be an ESE site by ESEfinder3.0 (Supplementary Figure S3) (37,38). This result was in agreement with previous studies using other chemically modified SSOs (42,43,44). Thus, splicing acceptor sites, splicing donor sites and ESE motifs are good targets for modulating exon splicing by LNA SSOs.

Second, we showed that exon skipping activity is dependent on the number of LNAs in the sequence of SSOs. Among the 15-mer mixmer SSOs, SSOs containing between five and eight LNA units showed especially high activity, and there was a correlation between their activity and the *T*_m_ of the SSOs with complementary RNA (Figure 3). In comparison to the LNA SSOs, the 2' -OMe SSO (five 2' -OMe and ten DNA) scarcely produced exon skipping (Figure 3), possibly because the exon skipping activity may be related to the binding affinity of each analogue. LNA-based oligonucleotides significantly enhanced hybridization to the complementary RNA, and the *T*_m_ was increased by 2ºC–8ºC for each LNA nucleotide incorporated (45,46), whereas the 2' -OMe modification resulted in an increase of only ∼1ºC per nucleotide incorporated (47). UV melting experiments performed under low-sodium conditions (10 mM phosphate buffer (pH 7.2) containing 10 mM NaCl) revealed that the *T*_m_ values of LNA SSO (five LNA and ten DNA) and 2' -OMe SSO (five 2' -OMe and ten DNA) were 69.5ºC and 50.1ºC, respectively (Supplementary Table S4). Thus, it seems that a higher melting temperature is associated with higher SSO activity. However, SSOs fully modified with LNA showed low activity despite their high *T*_m_ values. LNA-modified oligonucleotides often form stable self-structures (hairpin or self-dimer) (48). In particular, fully modified LNA SSOs might impair skipping activity because of self-dimerization, which led to decrease of effective SSO concentration for mRNA targeting (data not shown). In addition, we and others previously reported that AONs that possess very high binding affinity exhibit a relatively weak silencing ability (49,50). One of the reasons for this is that because AONs dissociated from the RNase H-dependent cleaved target mRNA could enter a new round of catalysis, LNA AONs with too high affinity might reduce the dissociation rate. Thus, LNA AONs are thought to require optimal binding affinity for efficient turnover activities in antisense reaction (51,52). Indeed, higher antisense effects were obtained by using LNA AONs whose *T*_m_ values were less than 65ºC (10 mM phosphate buffer (pH 7.2) containing 100 mM NaCl) (49,50). In the case of exon skipping, SSO is also thought to be recycled after dissociation from the excised region (53). Thus, the values are different from those obtained for AONs, and there may be an optimum *T*_m_ range to design effective SSOs that incorporate LNA. To date, few studies have shown that LNA can be used in SSOs both *in vivo* and *in vitro* (17,18,19,20,21,22), and no information is available regarding the effects of the position and number of LNAs. Here, we report for the first time that SSOs fully modified with LNA have lower activity than LNA/DNA mixmer SSOs. This result may at least partially be explained by the optimization of *T*_m_ values described above and/or by the kinetics of duplex formation between LNA-based SSOs and RNA (54). Christensen reported that the rate of association of full-length LNA-based 10-mer oligonucleotides to complementary RNA was lower than that of a LNA/DNA mixmer (five LNA and five DNA) and the association constant of the full-length LNA-based oligonucleotide to complementary RNA was 1.5- to 2-fold less than that of the LNA/DNA mixmer in the presence of magnesium ions. Therefore, SSOs fully modified with LNA may be too rigid to use as splicing modulators, in contrast to LNA/DNA mixmers.

Third, our study of SSO length indicates that optimal lengths exist for LNA SSOs to modulate splicing. The quantitative real-time RT-PCR results indicated that the 13-mer SSO showed the highest effectiveness for exon skipping, whereas exon skipping activities of the above 15-mer LNA SSOs were decreased with increasing length (Figure 4). In this experiment, we used SSOs in which a LNA monomer was introduced on every other base. Therefore, longer SSOs exhibited higher binding abilities with smaller *K*_d_ values (Supplementary Table S6). These results are in good agreement with the *T*_m_ values. Thus, the decreased exon skipping activity should be brought by the other reason, such as intra- or intermolecular structures due to high number of LNAs in the SSO sequence (see above). On the other hand, in these experiments, all SSOs had a PS backbone in their sequence to provide nuclease resistance (55). Although the PS backbone decreases the *T*_m_ by ∼1ºC per substitution (56,57), the PS-LNA oligonucleotides have still high *T*_m_ values due to high affinity of LNA for the complementary RNA (Supplementary Tables S4 and S5) (58). Thus, PS backbone did not influence the binding affinity of PS-LNA SSO toward complementary RNA very much. Taken together, these ideas may explain why shorter LNA SSOs showed high splicing activity, and why 13-mer LNA/DNA SSO mixmers had the highest effectiveness for exon skipping.

Although Ittig *et al.* showed that a 9-mer fully modified LNA SSO has no significant exon skipping effect (20), we here demonstrated, for the first time, that LNA SSOs as short as 7 mers have the potential to modulate splicing in a concentration-dependent manner provided that they are highly modified and display high *T*_m_ values (Figures 5 and 6). Shorter SSOs may have a further advantage in that the production costs of oligonucleotide drugs are higher than that of small molecules; therefore, shorter oligonucleotides may provide a cost-effective solution to the development of oligonucleotide drugs. Although, the activity of the 9-mer SSO (four LNA and five DNA) was weaker than that of the 13-mer SSO, quantitative real-time RT-PCR experiments revealed that the expression of skipped mRNA was similar among 9-, 19-, 21- and 23-mer SSOs (Figure 4). Intriguingly, when we compared two 9-mer LNA/DNA mixmer SSOs containing seven LNA analogues at different positions in each sequence, one of them presented 1.5-fold higher activity than the other despite their similar *T*_m_ values (87.1ºC and 83.1°C) (Supplementary Table S7). Thus, the position of LNA analogues in the SSO sequence may be an important factor for exon skipping. Of note, the 9-mer 2' -OMe SSO (four 2' -OMe and five DNA), which has a low *T*_m_ value (40.0ºC), exhibited no exon skipping activity at all (Figure 5).

Kandimalla *et al.* reported that short oligonucleotides (9-mers) bind more specifically than longer oligonucleotides (such as 21-mers), possibly because longer oligonucleotides have a higher chance of binding to various target sequences containing up to two mismatches than do short oligonucleotides, given the sufficient *T*_m_ values of longer oligonucleotides for forming duplexes with mismatched sequences (59). Indeed, Guterstam *et al.* demonstrated that the 18-mer PS LNA/2’-OMe mixmers with four mismatches, including one LNA mismatch, induced exon skipping (17). On the other hand, Obad *et al.* reported that 8-mer LNAs, termed tiny LNAs, inhibit microRNA activity without off-target effects (60). In this study, we evaluated the sequence specificity of LNA/DNA mixmer SSOs by introducing mismatches. The 13-mer LNA SSO (+110-1_6/13) containing one LNA mismatch was able to induce exon skipping, while the exon skipping activity is abolished when one to three LNA mismatches are introduced in the center of the 9-mer LNA SSO (+114-1_7/9_2). Thus, the 9-mer LNA SSO improved mismatch discrimination in comparison with the 13-mer LNA SSO. However, in our *in silico* analysis, the number of target genes that have perfect match with 9-mer LNA SSO (+114-1_7/9_2) is far larger than that of the 13-mer LNA SSO (+110-1_6/13) (914 genes and 8 genes, respectively) (Table 1). Although the ability to discriminate between the matched and mismatched sequences is improved by shorter SSO, these results suggest that it is important to design LNA SSOs in consideration of off target-effects.

In conclusion, we found that the number of LNAs in the SSO sequence, the *T*_m_ of the SSOs and the length of the LNA SSOs are key factors for their activity. We also show for the first time that 7-mer LNA SSOs induce exon skipping. Our findings suggest that LNA SSO-mediated exon skipping may be an attractive therapeutic strategy for genetic diseases.

## SUPPLEMENTARY DATA

Supplementary Data are available at NAR Online.

SUPPLEMENTARY DATA
