# Supplementary material for: Design and evaluation of locked nucleic acid-based splice-switching oligonucleotides in vitro
Source: Nucleic Acids Res. 2014 Jun 16;42(12):8174–87. doi: 10.1093/nar/gku512 (PMC4081108; doi:10.1093/nar/gku512)
Supplement: SUPPLEMENTARY DATA [file supp_42_12_8174__index.html]

Design and evaluation of locked nucleic acid-based splice-switching oligonucleotides in vitro — Design and evaluation of locked nucleic acid-based splice-switching oligonucleotides in vitro — SUPPLEMENTARY DATA 

# Design and evaluation of locked nucleic acid-based splice-switching oligonucleotides *in vitro*

## SUPPLEMENTARY DATA

**Files in this Data Supplement:**

- SUPPLEMENTARY DATA
