# Supplementary material for: Design and evaluation of locked nucleic acid-based splice-switching oligonucleotides in vitro
Source: Nucleic Acids Res. 2014 Jun 16;42(12):8174–87. doi: 10.1093/nar/gku512 (PMC4081108; doi:10.1093/nar/gku512)

[Supplementary Data]

Design and evaluation of locked nucleic acid-based splice-switching oligonucleotides in vitro

Takenori Shimo[1], Keisuke Tachibana[1], Kiwamu Saito[1], Tokuyuki Yoshida[1,2], Erisa Tomita[3], Reiko Waki[1], Tsuyoshi Yamamoto[1], Takefumi Doi[1], Takao Inoue[1,2], Junji Kawakami[3,4] and Satoshi Obika[1,*]

[1] Graduate School of Pharmaceutical Sciences, Osaka University, 1-6, Yamadaoka, Suita, Osaka, 565-0871, Japan
[2] Division of Cellular and Gene Therapy Products, National Institute of Health Sciences, 1-18-1 Kamiyoga, Setagaya-ku, Tokyo 158-8501, Japan
[3] Department of Nanobiochemistry, FIRST, Konan University, 7-1-20 Minatojima-minamimachi, Chuo-ku, Kobe 650-0047, Japan
[4] Frontier Institute for Biomolecular Engineering Research (FIBER), Konan University, 7-1-20 Minatojima-minamimachi, Chuo-ku, Kobe 650-0047, Japan

* To whom correspondence should be addressed. Tel: +81 6 6879 8200; Fax: +81 6 6879 8204; Email: obika@phs.osaka-u.ac.jp

The authors wish it to be known that, in their opinion, the first two authors should be regarded as joint First Authors.

SUPPLEMENTARY MATERIAL AND METHODS

Thermodynamic analysis of various lengths of LNA/DNA mixmer SSOs duplexes

Gibbs free energy change ($\Delta G°_{25}$) during the duplex formation of 9-mer SSO (+114-1_4/9) and the complementary RNA was calculated by standard thermal UV melting analysis (61). Dissociation constant (K_d) for the duplex was determined using the equation, $K_d = 1/\exp(-\Delta G°/298.15R)$, where R was gas constant. Because the binding of our LNA/DNA mixmer SSOs to mRNA was too strong to determine the dissociation constant directly from calorimetric or stopped flow experiments, $\Delta G°_{25}$ and dissociation constant at 25°C for the other SSOs were estimated by competition assays.

In our analysis, equal amount of a 5' 6-carboxyfluorescein (6-FAM)-labeled SSO (A strand) and the complementary RNA (B strand) was mixed with various amount of a competitor SSO (X strand) in 10 mM phosphate buffer (pH 7.2) containing 100 mM NaCl. The initial concentration of each (A and B) strand was C_0 (6.67×10^{-8} M). To give equal opportunities to the competing strands (A and X) for hybridization with RNA (B strand), B strand was added to the solution in last. The solution was heated to 100°C and annealed to 25°C with cooling rate 0.5°C/min. After separation by 15% non-denaturing PAGE, the gel was analyzed using an ImageQuant LAS4010 (GE Healthcare Bio-sciences AB, Uppsala, Sweden). The fluorescence intensity that corresponds to the amount of a labeled duplex (F_{AB}) was measured by using ImageJ software. The decrement of the F_{AB} with increasing free competitor SSO concentration [X] was fitted to the following equation with an assumption [A] >> $K_{d(AB)}$, where Max and BG respectively indicated maximum change of F_{AB} and background fluorescence intensity, and half maximal inhibitory concentration of the competitor SSO (IC_{50}) was determined (Supplementary Figure S11).

$$F_{AB} = Max(1 - [X]/([X] + IC_{50})) + BG$$

In this analysis, IC_{50} is defined as the concentration of free competitor SSO at which half of the labeled duplex is present at equilibrium. The analysis was carried out at least three times independently and the average IC_{50} values were adopted. The relative affinities between two competing molecules were calculated as follows.

$$K_{d(BX)}/K_{d(AB)} = 2 \times IC_{50}/C_0$$

$$\Delta\Delta G°_{25} = \Delta G°_{25(BX)} - \Delta G°_{25(AB)} = 298.15R \ln(K_{d(BX)}/K_{d(AB)})$$

Based on the resulting $\Delta\Delta G°_{25}$ and actual measured $\Delta G°_{25}$ value for 9-mer SSO (+114-1_4/9), $\Delta G°_{25}$ and K_d values for the other SSOs were estimated.

SUPPLEMENTARY TABLE AND FIGURES LEGENDS

Supplementary Table S1. SSOs used for the first screening. Nine SSOs for dystrophin exon 58 skipping are shown. Sequences are shown from 5' to 3'. Capital letter A, G, T: LNA; C: 5-methyl cytosine LNA; lowercase letter: DNA.

Supplementary Table S2. SSOs used for the second screening. Twenty-nine SSOs for dystrophin exon 58 skipping are shown. Sequences are shown from 5' to 3'.

Supplementary Table S3. SSOs used for the third screening. Ten SSOs for dystrophin exon 58 skipping are shown. Sequences are shown from 5' to 3'.

Supplementary Table S4. SSOs targeting 5' splice site used for T_m and exon skipping analysis. SSOs for each experiment are shown. Sequences are shown from 5' to 3'. T_m values (low salt: 2 μM duplex in 10 mM phosphate buffer (pH 7.2), 10 mM NaCl (n = 4); medium salt: 2 μM duplex in 10 mM phosphate buffer (pH 7.2), 100 mM NaCl (n = 3)) were determined (± SD). Capital letter A, G, T: LNA; C: 5-methyl cytosine LNA; capital letter with underline: 2'-OMe RNA; lowercase letter: DNA.

Supplementary Table S5. SSOs targeting 3' splice site used for T_m and exon skipping analysis. SSOs for each experiment are shown. Sequences are shown from 5' to 3'. T_m values (2 μM duplex in 10 mM phosphate buffer (pH 7.2), 10 mM NaCl) were determined in four independent experiments (± SD).

Supplementary Table S6. SSOs used for analysis of SSO length. SSOs for each experiment are shown. Sequences are shown from 5' to 3'. T_m values (2 μM duplex in 10 mM phosphate buffer (pH 7.2), 10 mM NaCl) were determined in four independent experiments (± SD). Gibbs free energy change ($\Delta G°_{25}$) for SSO (+114-1_4/9) was calculated by standard thermal UV melting analysis (2 μM duplex in 10 mM phosphate buffer (pH 7.2), 100 mM NaCl). Dissociation constant (K_d) for SSO (+114-

1_4/9) was calculated using the equation: $K_d = 1/\exp(-\Delta G°/298.15R)$. $\Delta G°_{25}$ and K_d for the other SSOs were estimated by competition assays.

Supplementary Table S7. SSOs used for analysis of 9-mer SSOs. SSOs for each experiment are shown. Sequences are shown from 5' to 3'. T_m values (2 μM duplex in 10 mM phosphate buffer (pH 7.2), 10 mM NaCl) were determined in four independent experiments (± SD).

Supplementary Table S8. SSOs used for analysis of short SSOs. SSOs for each experiment are shown. Sequences are shown from 5' to 3'. T_m values (2 μM duplex in 10 mM phosphate buffer (pH 7.2), 10 mM NaCl) were determined in three or four independent experiments (± SD).

Supplementary Table S9. SSOs used for mismatch discrimination. SSOs for each experiment are shown. Sequences are shown from 5' to 3'. Nucleotides of mismatch sites are double underlined.

Supplementary Table S10. Primers used for RT-PCR analysis. Sequences of forward (For.) and reverse (Rev.) primer for each target are shown. Sequences are shown from 5' to 3'.

Supplementary Table S11. Primers used for quantitative real-time RT-PCR analysis. Sequences of forward (For.) and reverse (Rev.) primer for each target are shown. Sequences are shown from 5' to 3'.

Supplementary Figure S1. Schematic representation of the dystrophin reporter minigene and of the its splicing pattern. Human dystrophin exons are indicated by open boxes and introns by narrow lines. Solid boxes represent vector sequences. Lines connecting the exons represent the splicing patterns. The expected mRNA structures, indicated below the minigene structure result from inclusion or exclusion of exon 58. Small black arrows and small purple arrows indicate approximate positions of primers used for RT-PCR and quantitative real-time RT-PCR, respectively.

Supplementary Figure S2. Screening of LNA SSOs designed to induce dystrophin exon 58 skipping. **(A, B)** Reporter cells were transfected with the indicated SSOs (100 nM) for 24 h. The levels of reporter minigene mRNA fragments were measured by RT-PCR, and the signal intensity of each band was normalized according to its nucleotide composition. The exon skipping percentage was calculated as the amount of exon skipped transcript relative to the total amount of exon skipped and full-length transcripts. Values represent the mean ± standard deviation of triplicate or sixplicate samples. Reproducible results were obtained from two independent experiments. (A) and (B) express the results of the first and the second screening, respectively. Mock: treated with Lipofectamine only; no treatment: no transfection.

Supplementary Figure S3. Prediction of splice factor binding site in human dystrophin exon 58. The locations of potential binding sites for the splicing factors SRSF1 (SF2/ASF), SRSF1 (IgM-BRCA1), SRSF2 (SC35), SRSF5 (SRp40), and SRSF6 (SRp55) in human dystrophin exon 58 and 50 bases of flanking intronic sequence were predicted by using ESEfinder3.0.

Supplementary Figure S4. Effect of the number of LNA variations on exon skipping activity. **(A, B)** Reporter cells were transfected with the indicated SSOs (30 nM), targeting the 5' (A) or 3' (B) splice site, for 24 h. The levels of reporter minigene mRNA fragments were measured by RT-PCR, and the signal intensity of each band was normalized according to its nucleotide composition. The exon skipping percentage was calculated as the amount of exon skipped transcript relative to the total amount of exon skipped and full-length transcripts. LNA SSO (+10+24), which showed no exon skipping effects, was used as a control. Values represent the mean ± standard deviation of triplicate samples. Reproducible results were obtained from two independent experiments. The T_m of each SSO with a complementary RNA under low-sodium conditions is also shown. # indicates that no sigmoidal melting curve was observed, even at higher T_m values. The data are the mean ± standard deviation (n = 4). Mock: treated with Lipofectamine only; no treatment: no transfection.

Supplementary Figure S5. Assessment of the effect of the length of LNA/DNA mixmer SSOs on exon skipping. Reporter cells were transfected with the indicated SSOs (30 nM) for 24 h. The levels of reporter minigene mRNA fragments were measured by RT-PCR, and the signal intensity of each band was normalized according to its nucleotide composition. The exon skipping percentage was calculated as the amount of exon skipped transcript relative to the total amount of exon skipped and full-length transcripts. LNA SSO (+10+24), which showed no exon skipping effects, was used as a control. Values represent the mean ± standard deviation of triplicate samples. Reproducible results were obtained from two independent experiments. The T_m of each SSO with a complementary RNA under low-sodium conditions is also shown. # indicates that no sigmoidal melting curve was observed, even at higher T_m values. The data are the mean ± standard deviation (n = 4). Mock: treated with Lipofectamine only; no treatment: no transfection.

Supplementary Figure S6. Exon skipping activity of LNA/DNA mixmer SSOs at various concentrations. **(A, C)** Reporter cells were transfected with the indicated SSOs, targeting the 3' (A) or 5' (C) splice site, at various concentrations for 24 h. The levels of reporter minigene mRNA fragments were measured by RT-PCR, and the signal intensity of each band was normalized according to its nucleotide composition. The exon skipping percentage was calculated as the amount of exon skipped transcript relative to the total amount of exon skipped and full-length transcripts. LNA SSO (+10+24), which showed no exon skipping effects, was used as a control. **(B)** RT-PCR analysis shows the full-length upper band (587 bp) and the skipped lower band (466 bp). GAPDH was used as an internal control. **(D)** The exon 58 skipping levels were measured by quantitative real-time RT-PCR and normalized to GAPDH mRNA levels, relative to the values in the mock set as 1. Values represent the mean ± standard deviation of triplicate samples. Reproducible results were obtained from two independent experiments. Mock: treated with Lipofectamine only; no treatment: no transfection.

Supplementary Figure S7. Inducing exon skipping by 9-mer LNA/DNA mixmer SSOs. Reporter cells were transfected with the indicated SSOs (30 nM) for 24 h. The levels of reporter minigene mRNA fragments were measured by RT-PCR and the signal intensity of each band was normalized according to its nucleotide composition. The exon skipping percentage was calculated as the amount of exon skipped transcript relative to the total amount of exon skipped and full-length transcripts. LNA

SSO (+10+24), which showed no exon skipping effects, was used as a control. Values represent the mean ± standard deviation of triplicate samples. Reproducible results were obtained from two independent experiments. The T_m of each SSO with a complementary RNA under low-sodium conditions is also shown. The data are the mean ± standard deviation (n = 4). Mock: treated with Lipofectamine only; no treatment: no transfection.

Supplementary Figure S8. 9-mer LNA SSOs induce exon skipping in a concentration-dependent manner. **(A-D)** RT-PCR analyses of RNA samples from reporter cells treated with the indicated SSOs at various concentrations for 24 h show the full-length upper band (587 bp) and the skipped lower band (466 bp). LNA SSO (+10+24), which showed no exon skipping effects, was used as a control. GAPDH was used as an internal control. **(E)** The signal intensity of each band was normalized according to its nucleotide composition. The exon skipping percentage was calculated as the amount of exon skipped transcript relative to the total amount of exon skipped and full-length transcripts. Values represent the mean ± standard deviation of triplicate samples. Reproducible results were obtained from two independent experiments. Mock: treated with Lipofectamine only; no treatment: no transfection.

Supplementary Figure S9. Exon skipping activity of short (6- to 9-mer) LNA SSOs. RT-PCR analyses of RNA samples from reporter cells treated with the indicated SSOs (30 nM) for 24 h. The signal intensity of each band was normalized according to its nucleotide composition. The exon skipping percentage was calculated as the amount of exon skipped transcript relative to the total amount of exon skipped and full-length transcripts. LNA SSO (+10+24), which showed no exon skipping effects, was used as a control. GAPDH was used as an internal control. Values represent the mean ± standard deviation of triplicate samples. Reproducible results were obtained from two independent experiments. The T_m of each SSO with a complementary RNA under low-sodium conditions is also shown. The data are the mean ± standard deviation (n = 3-4). Mock: treated with Lipofectamine only; no treatment: no transfection.

Supplementary Figure S10. Effect of mismatches on exon skipping activity and specificity. Reporter cells were transfected with the indicated SSOs (30 nM) for 24 h. The levels of reporter minigene mRNA fragments were measured by RT-PCR, and the signal intensity of each band was normalized according to its nucleotide composition. The exon skipping percentage was calculated as the amount of exon skipped transcript relative to the total amount of exon skipped and full-length transcripts. LNA SSO (+10+24), which showed no exon skipping effects, was used as a control. Values represent the mean ± standard deviation of triplicate samples. Reproducible results were obtained from two independent experiments. Mock: treated with Lipofectamine only; no treatment: no transfection.

Supplementary Figure S11. Quantitative analysis of competitive inhibition of 5' 6-FAM-labeled SSO duplex formation to the complementary RNA. Equal amount of a 5' 6-FAM-labeled SSO (A strand) and the complementary RNA (B strand) was mixed with various amount of an unlabeled competitor SSO (X strand) and annealed in 10 mM phosphate buffer (pH 7.2) containing 100 mM NaCl. After separation by 15% non-denaturing PAGE, the decrement of the fluorescence intensity that

corresponds to the amount of a labeled duplex (AB signal) with increasing free competitor SSO concentration [X] was curve fitted and half maximal inhibitory concentration of the competitor SSO (IC_{50}) was determined.

Supplementary Table S1.

Entry	ID	Sequence
1	-5+10	tCt tGa aGg cCt gTg
2	+10+24	aGt tTt cAa tTc cCt
3	+25+39	gAt tAc aGg tTc tTt
4	+40+54	cTc aAg aGt aCt cAt
5	+55+69	aAa tAt tCg tAc aGt
6	+70+84	aGg cTg cTc tGt cAg
7	+85+99	cTc tAg tCc tTc cAa
8	+100+114	cTc cTg gTa gAg tTt
9	+115-8	tCa aTt aCc tCt gGg

Supplementary Table S2.

Entry	ID	Sequence
1-1	-20-6	aAa tGa gAt gAa aAg
1-2	-17-3	gTg aAa tGa gAt gAa
1-3	-14+1	cCt gTg aAa tGa gAt
1-4	-11+4	aGg cCt gTg aAa tGa
1-5	-8+7	tGa aGg cCt gTg aAa
1	-5+10	tCt tGa aGg cCt gTg
1-6	-2+13	cCc tCt tGa aGg cCt
1-7	+2+16	aTt cCc tCt tGa aGg
1-8	+5+19	tCa aTt cCc tCt tGa
1-9	+8+22	tTt tCa aTt cCc tCt
6-1	+58+72	cAg aAa tAt tCg tAc
6-2	+61+75	tGt cAg aAa tAt tCg
6-3	+64+78	cTc tGt cAg aAa tAt
6-4	+67+81	cTg cTc tGt cAg aAa
6	+70+84	aGg cTg cTc tGt cAg
6-5	+73+87	cAa aGg cTg cTc tGt
6-6	+76+90	tTc cAa aGg cTg cTc
6-7	+79+93	tCc tTc cAa aGg cTg
6-8	+82+96	tAg tCc tTc cAa aGg
9-1	+103+117	gGg cTc cTg gTa gAg
9-2	+106+120	tCt gGg cTc cTg gTa
9-3	+109-2	aCc tCt gGg cTc cTg
9-4	+112-5	aTt aCc tCt gGg cTc
9	+115-8	tCa aTt aCc tCt gGg
9-5	+118-11	cAt tCa aTt aCc tCt
9-6	+121-14	cCa cAt tCa aTt aCc
9-7	-3-17	gTt cCa cAt tCa aTt
9-8	-6-20	aTa gTt cCa cAt tCa
9-9	-9-23	aTt aTa gTt cCa cAt

Supplementary Table S3.

Entry	ID	Sequence
1-6-1	-4+11	ctC ttG aaG gcC tgT
1-6-2	-3+12	Cct Ctt Gaa Ggc Ctg
1-6	-2+13	cCc tCt tGa aGg cCt
1-6-3	-1+14	tcC ctC ttG aaG gcC
1-6-4	+1+15	Ttc Cct Ctt Gaa Ggc
9-2-1	+104+118	tgG gcT ccT ggT agA
9-2-2	+105+119	Ctg Ggc Tcc Tgg Tag
9-2	+106+120	tCt gGg cTc cTg gTa
9-2-3	+107+121	ctC tgG gcT ccT ggT
9-2-4	+108-1	Cct Ctg Ggc Tcc Tgg

Supplementary Table S4.

Entry	ID	Sequence	T_{m} (ºC) Low salt	T_{m} (ºC) Medium salt
1	-1+14_15/15	TCC CTC TTG AAG GCC	>95.0	–
2	-1+14_8/15	TcC cTc TtG aAg GcC	82.6 ± 2.3	–
3	-1+14_7/15	tCc CtC tTg AaG gCc	84.6 ± 2.7	–
4	-1+14_5/15	tcC ctC ttG aaG gcC	69.5 ± 0.5	79.1 ± 0.2
5	-1+14_4/15	tcC ctC ttG aaG gcC	68.8 ± 2.6	74.8 ± 1.2
6	-1+14_3/15	tcC ctC ttG aaG gcC	62.1 ± 1.7	70.2 ± 1.2
7	-1+14_2/15	tcC ctC ttG aaG gcC	57.7 ± 2.1	66.1 ± 1.0
8	-1+14_1/15	tcC ctC ttG aaG gcC	55.1 ± 1.5	63.3 ± 0.2
9	-1+14_OMe	tcC ctC ttG aaG gcC	50.1 ± 1.8	58.4 ± 0.4
10	-1+14_DNA	tcc ctc ttg aag gcc	39.9 ± 1.3	52.2 ± 1.1

Supplementary Table S5.

Entry	ID	Sequence	T_m (ºC)
1	+108-1_15/15	CCT CTG GGC TCC TGG	>95.0
2	+108-1_8/15	CcT cTg GgC tCc TgG	>88.0
3	+108-1_7/15	cCt CtG gGc TcC tGg	87.8 ± 0.6
4	+108-1_5/15	Cct Ctg Ggc Tcc Tgg	79.0 ± 1.9
5	+108-1_4/15	Cct Ctg Ggc Tcc Tgg	73.8 ± 0.8
6	+108-1_3/15	Cct Ctg Ggc Ucc Tgg	66.8 ± 0.4
7	+108-1_2/15	Cct Ctg Ggc Ucc Tgg	63.8 ± 1.0
8	+108-1_1/15	Cct Ctg Ggc Ucc Ugg	61.7 ± 0.2
9	+108-1_OMe	Cct Ctg Ggc Ucc Ugg	57.4 ± 0.8
10	+108-1_DNA	cct ctg ggc tcc tgg	53.0 ± 1.0

Supplementary Table S6.

Entry	ID	Sequence	T_m (ºC)	$\Delta G°_{25}$ (kcal/mol)	K_d (M)
1	+114-1_4/9	cCt CtG gGc	66.7 ± 0.4	-20.7 ± 0.2	6.9×10^{-16}
2	+112-1_5/11	cCt CtG gGc Tc	75.8 ± 0.7	-23.9	3.2×10^{-18}
3	+110-1_6/13	cCt CtG gGc TcC t	83.3 ± 0.7	-27.2	1.2×10^{-20}
4	+108-1_7/15	cCt CtG gGc TcC tGg	87.8 ± 0.6	-29.2	3.7×10^{-22}
5	+106-1_8/17	cCt CtG gGc TcC tGg Ta	>88.0	-29.4	2.6×10^{-22}
6	+104-1_9/19	cCt CtG gGc TcC tGg TaG a	>88.0	-29.7	1.8×10^{-22}
7	+102-1_10/21	cCt CtG gGc TcC tGg TaG aGt	>88.0	-29.5	2.3×10^{-22}
8	+100-1_11/23	cCt CtG gGc TcC tGg TaG aGt Tt	>88.0	-30.0	1.1×10^{-22}

Supplementary Table S7.

Entry	ID	Sequence	T_m (ºC)
1	+114-1_4/9	cCt CtG gGc	66.7 ± 0.4
2	+114-1_7/9_1	cCT CTG GGc	87.1 ± 1.9
3	+114-1_7/9_2	CCT CTG gGc	83.1 ± 2.7
4	+114-1_OMe	c<u>C</u>t <u>C</u>t<u>G</u> g<u>G</u>c	40.0 ± 1.0

Supplementary Table S8.

Entry	ID	Sequence	T_m (ºC)
1	+114-1_9/9	`CCT CTG GGC`	87.0 ± 2.2
2	+114-1_7/9_2	`CCT CTG gGc`	83.1 ± 2.7
3	+114-1_4/9	`cCt CtG gGc`	66.7 ± 0.4
4	+115-1_8/8	`CCT CTG GG`	76.3 ± 1.5
5	+115-1_6/8	`CCT CTg Gg`	72.1 ± 0.4
6	+115-1_4/8	`cCt CtG gG`	61.0 ± 1.6
7	+116-1_7/7	`CCT CTG G`	72.8 ± 1.4
8	+116-1_5/7	`CCT CtG g`	57.7 ± 2.3
9	+116-1_3/7	`cCt CtG g`	47.9 ± 0.7
10	+117-1_6/6	`CCT CTG`	53.4 ± 2.8
11	+117-1_4/6	`CCT cTg`	45.2 ± 2.3
12	+117-1_3/6	`cCt CtG`	37.1 ± 2.1

Supplementary Table S9.

Entry	ID	Sequence
1	+110-1_6/13	cCt CtG gGc TcC t
2	+110-1_G117A	cCt CtA gGc TcC t
3	+110-1_G117C	cCt CtC gGc TcC t
4	+110-1_G117T	cCt CtT gGc TcC t
5	+110-1_G115C/G117C	cCt CtC gCc TcC t
6	+110-1_G115C/g116c/G117C	cCt CtC cCc TcC t
7	+114-1_7/9_2	CCT CTG gGc
8	+114-1_T118A	CCT CAG gGc
9	+114-1_T118C	CCT CCG gGc
10	+114-1_T118G	CCT CGG gGc
11	+114-1_G117C/C119G	CCT GTC gGc
12	+114-1_G117C/T118C/C119G	CCT GCC gGc

Supplementary Table S10.

Gene		Sequence	Size (bp)
Dystrophin reporter minigene	For. primer:	AACGGTACCAACGCTGCTGTTCTTTTTCA	587 (Exon 58 inclusion)
	Rev. primer:	CTTGGAGCCGTACTGGAACT	466 (Exon 58 skipping)
DMD	For. primer:	AAACAATGGCAAGACCTCCA	
	Rev. primer:	CTGGACAGACGTGGAAAGAAA	
	Nested For. primer:	GTCTCTCAACATTAGGTCCCATTT	575 (Exon 58 inclusion)
	Nested Rev. primer:	CTCCTCGAAGTGCCTTGACT	454 (Exon 58 skipping)
GAPDH	For. primer:	ACCACAGTCCATGCCATCAC	452
	Rev. primer:	TCCACCACCCTGTTGCTGTA	

Supplementary Table S11.

Gene		Sequence	Size (bp)
DMD	For. primer:	GATGTACATAGGAGCTGCCTC	70
			(Exon 57/59 junction)
	Rev. primer:	TCAGCCTGCTTTCGTAGA	(Exon 59)
GAPDH	For. primer:	GGTCACCAGGGCTGCTTTT	85
	Rev. primer:	GTAAACCATGTAGTTGAGGTCAATGAAG	

SUPPLEMENTARY REFERENCES

61. Kawakami, J., Tanaka, Y. and Kishimoto, K. (2009) Accurate curve fitting procedure for UV melting analysis of highly thermostable RNA hairpins. *Nucleic Acids Symp Ser (Oxf)*, 227-228.

Supplementary Fig. 1

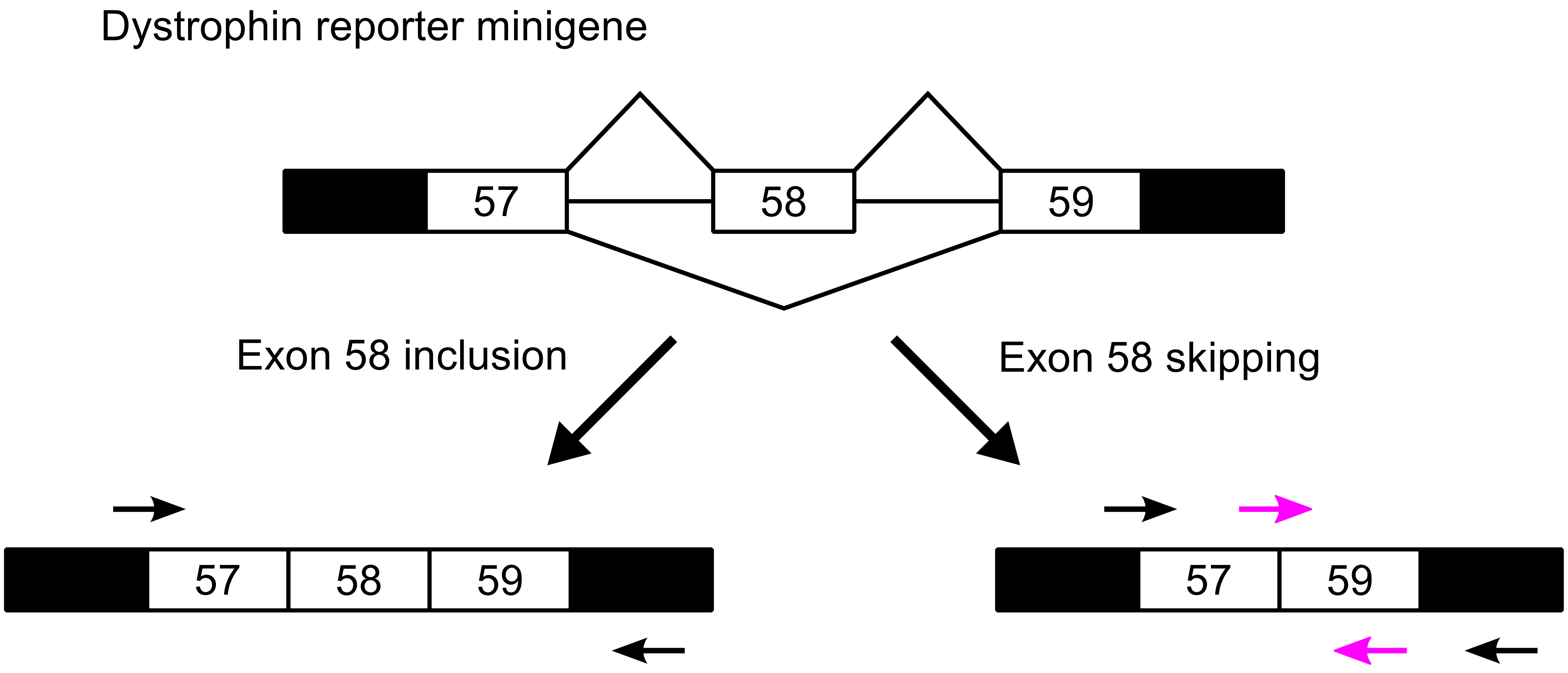

Supplementary Fig. 2

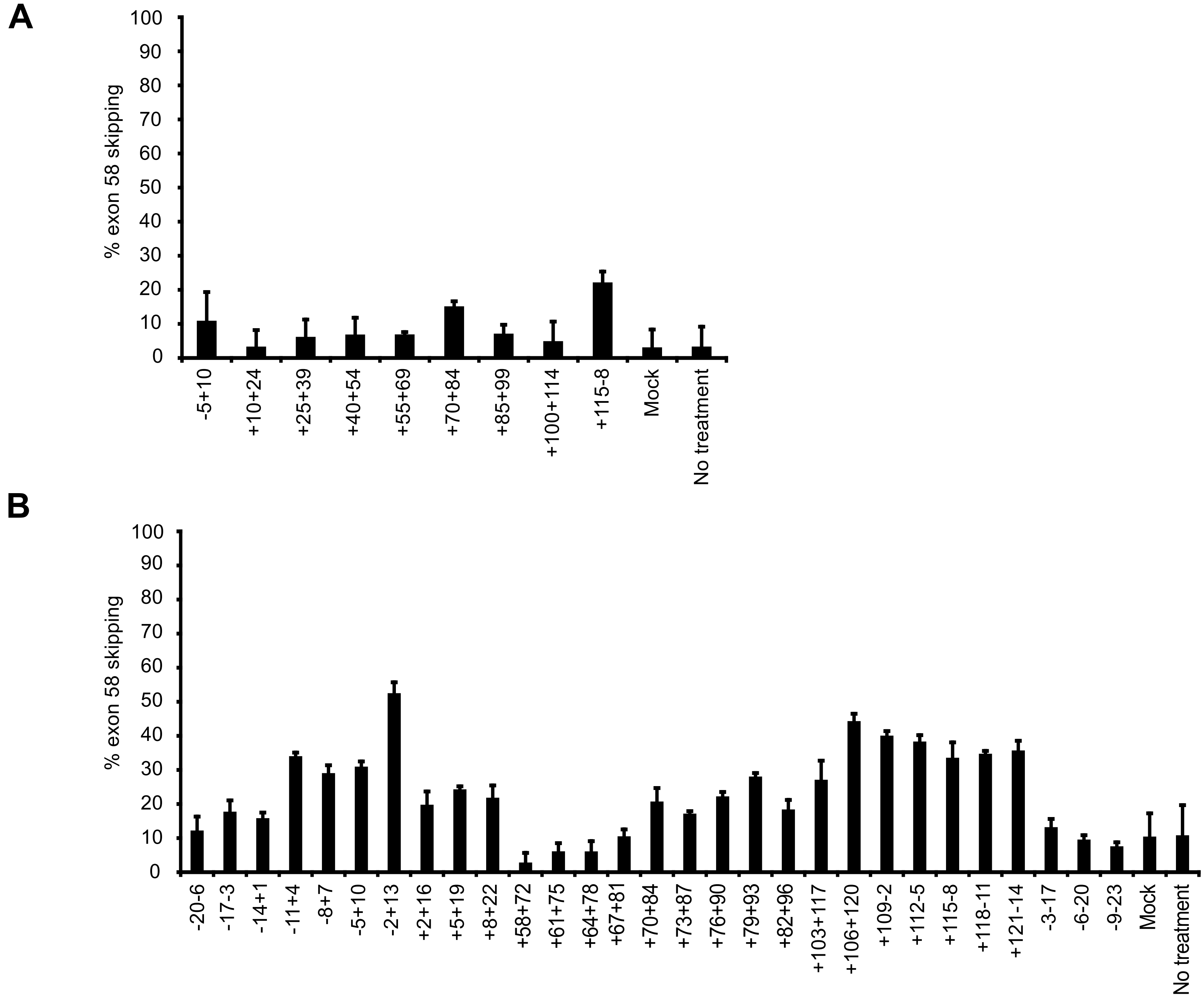

Supplementary Fig. 3

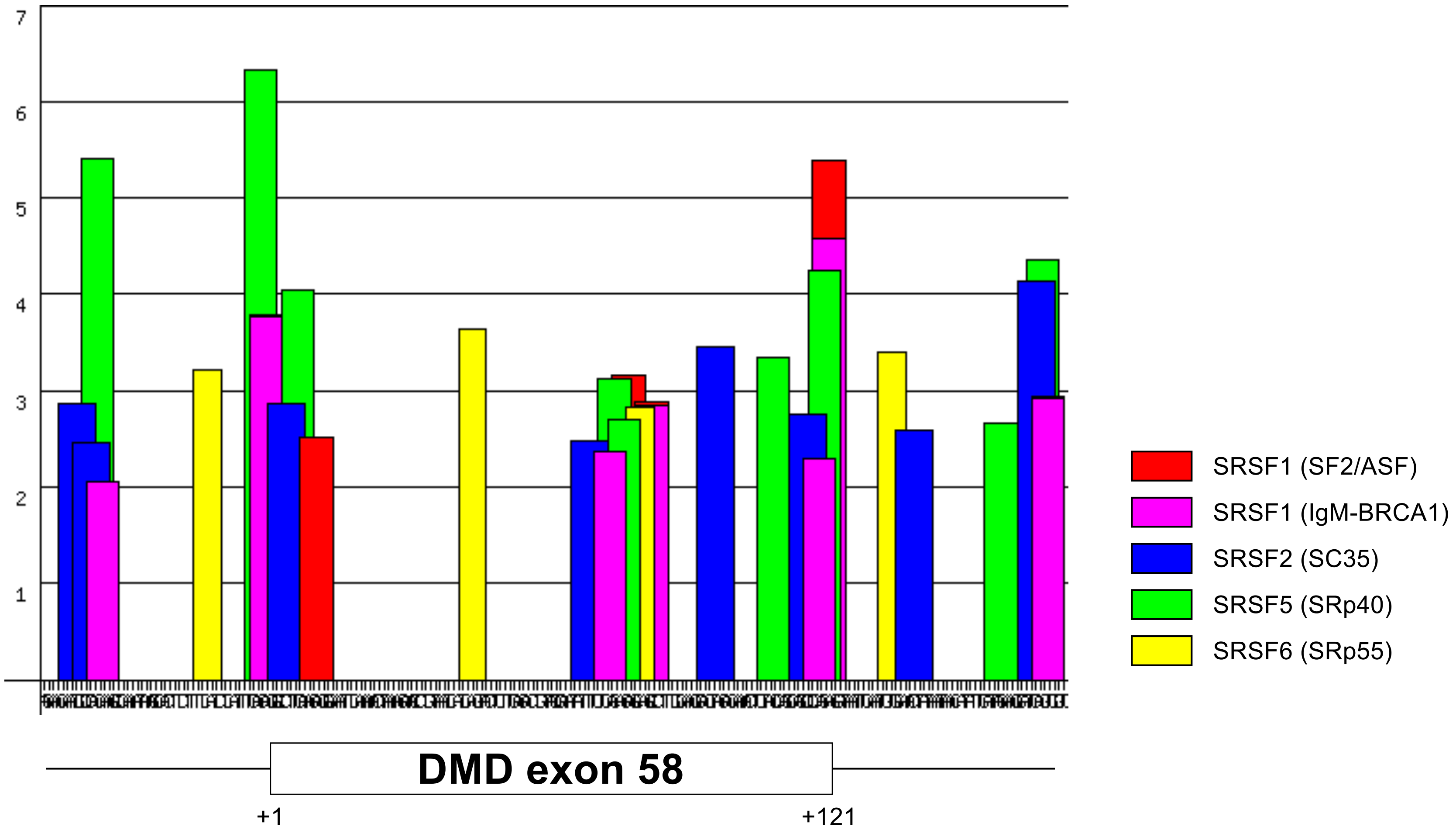

Supplementary Fig. 4

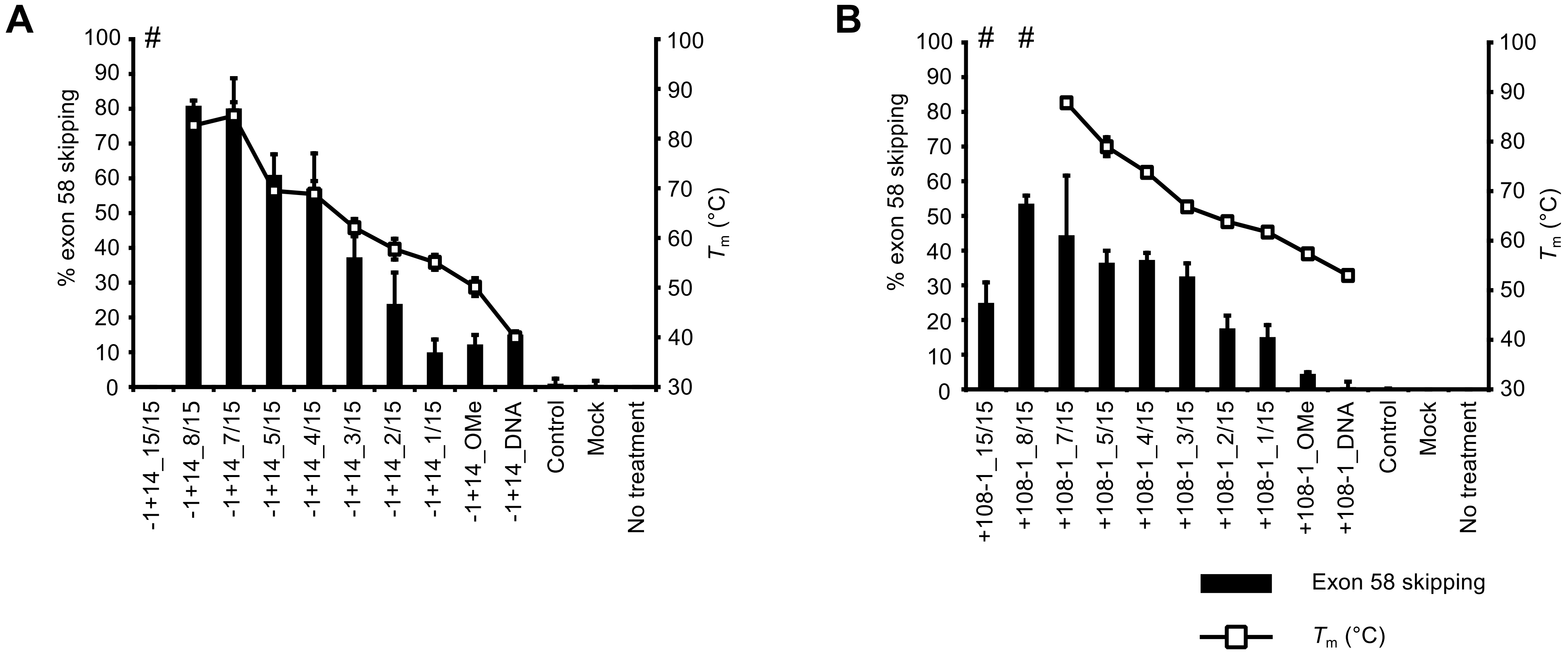

Supplementary Fig. 5

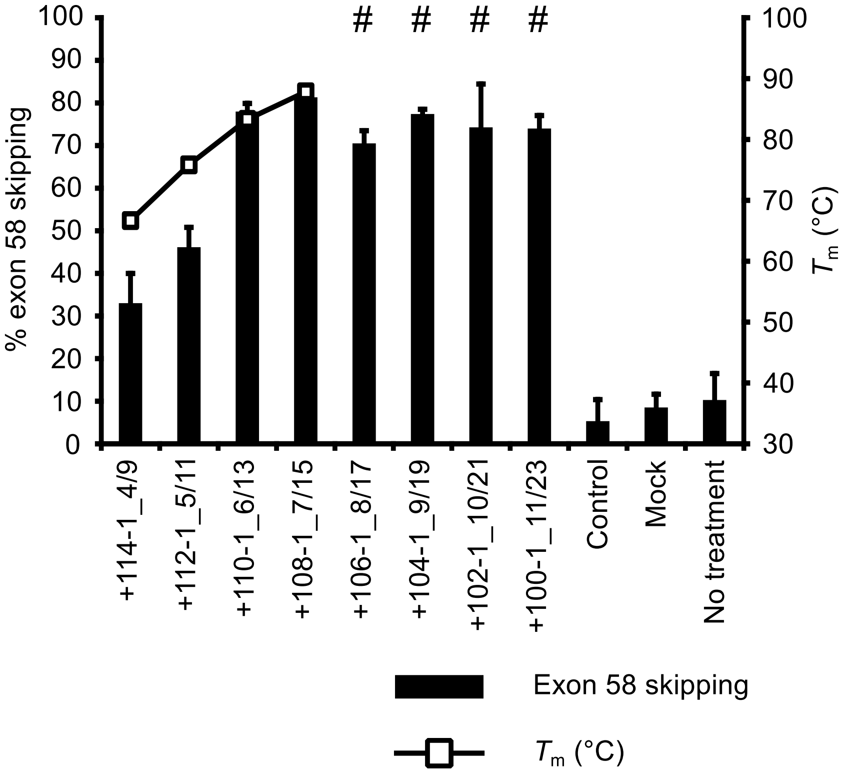

Supplementary Fig. 6

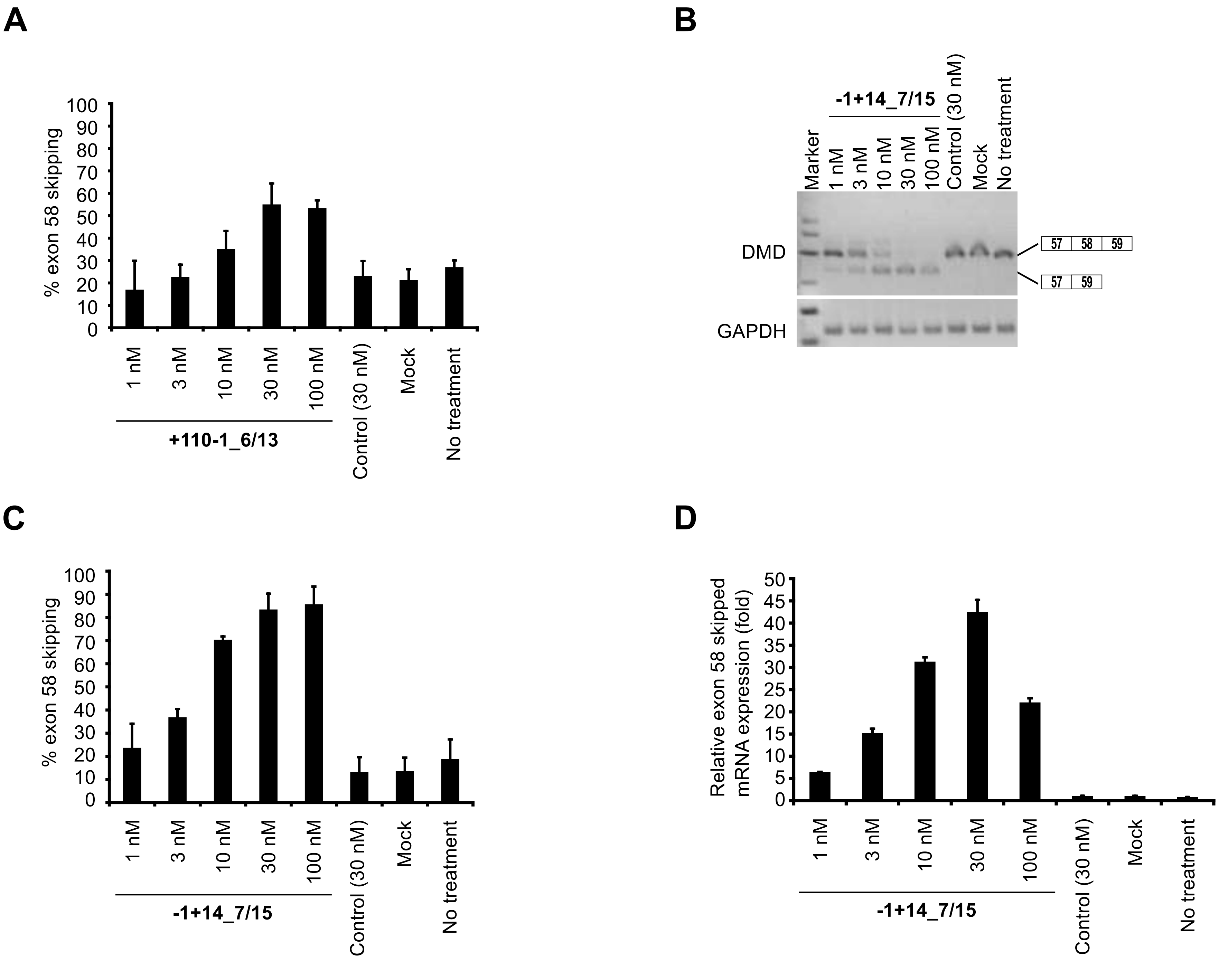

Supplementary Fig. 7

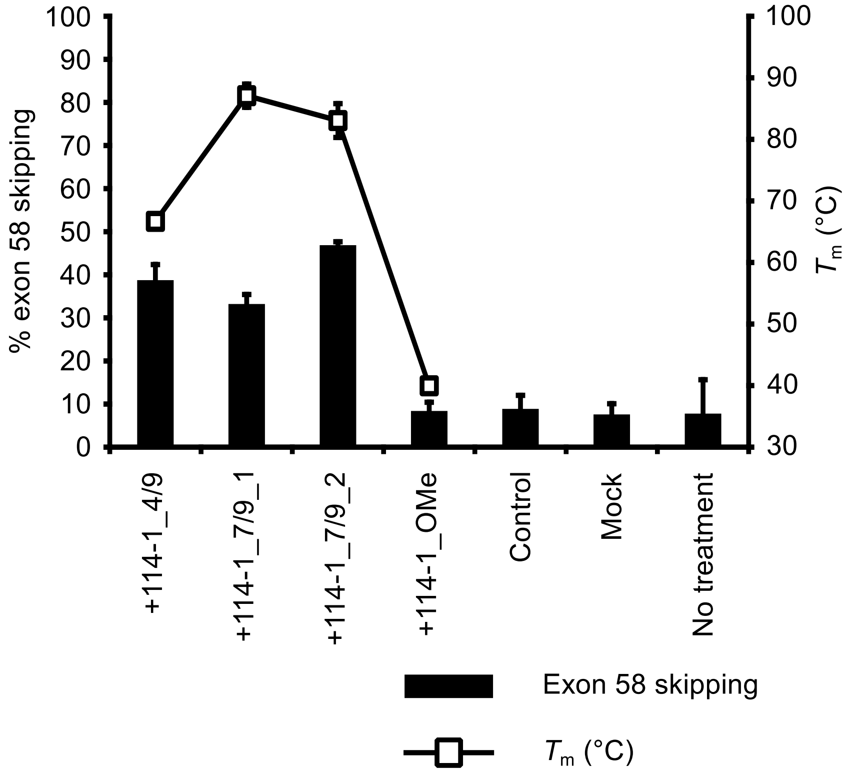

Supplementary Fig. 8

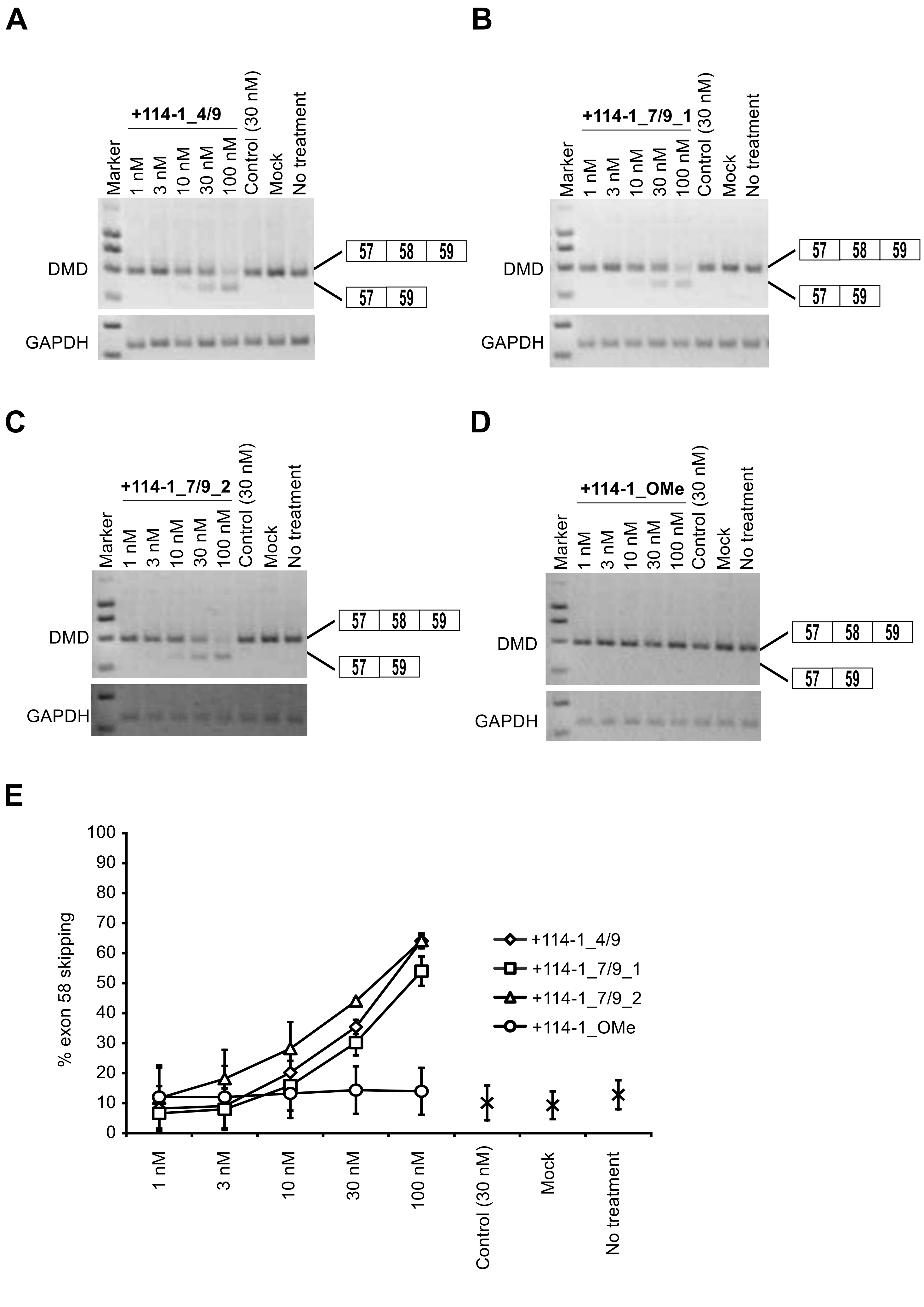

Supplementary Fig. 9

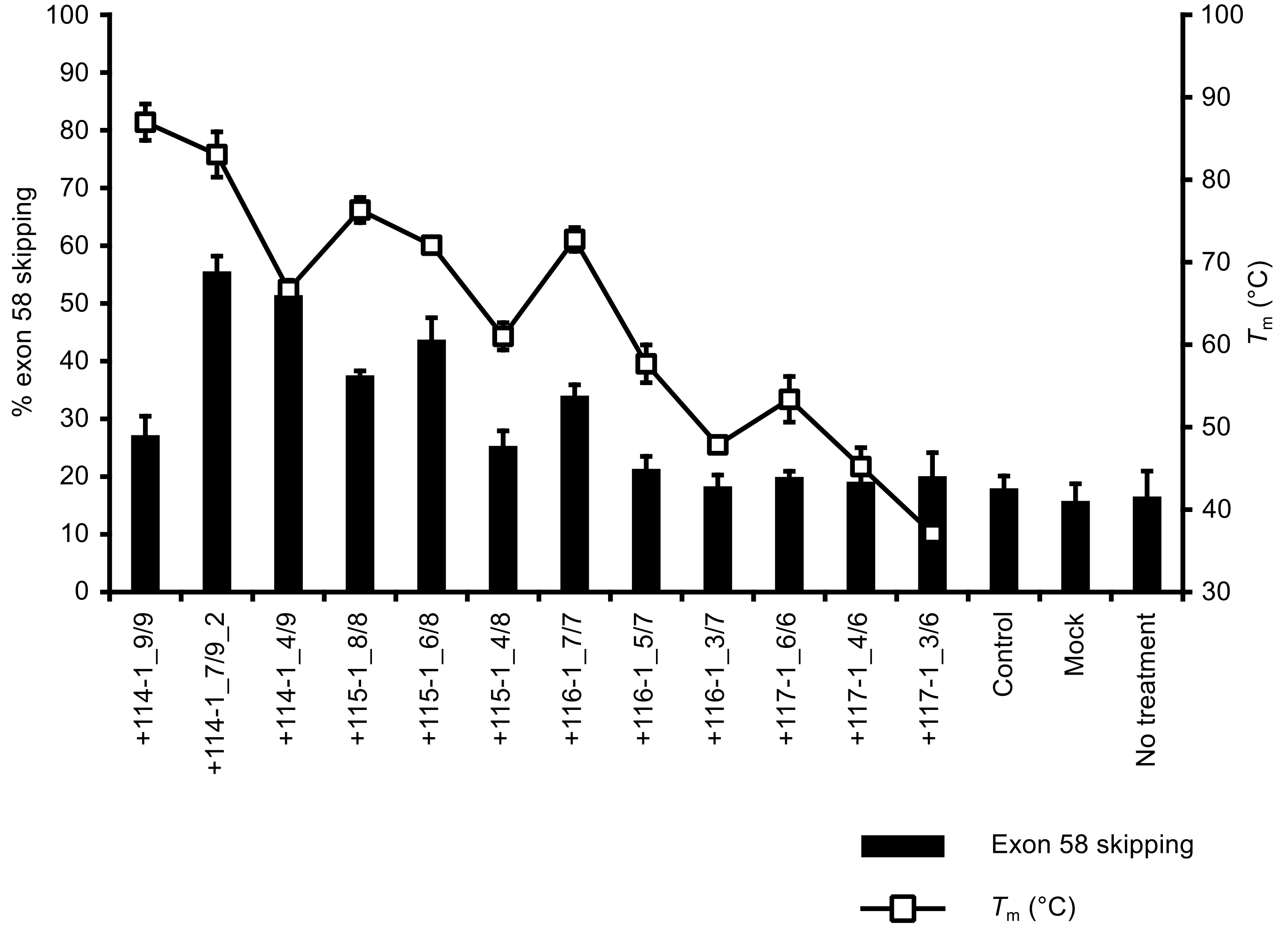

Supplementary Fig. 10

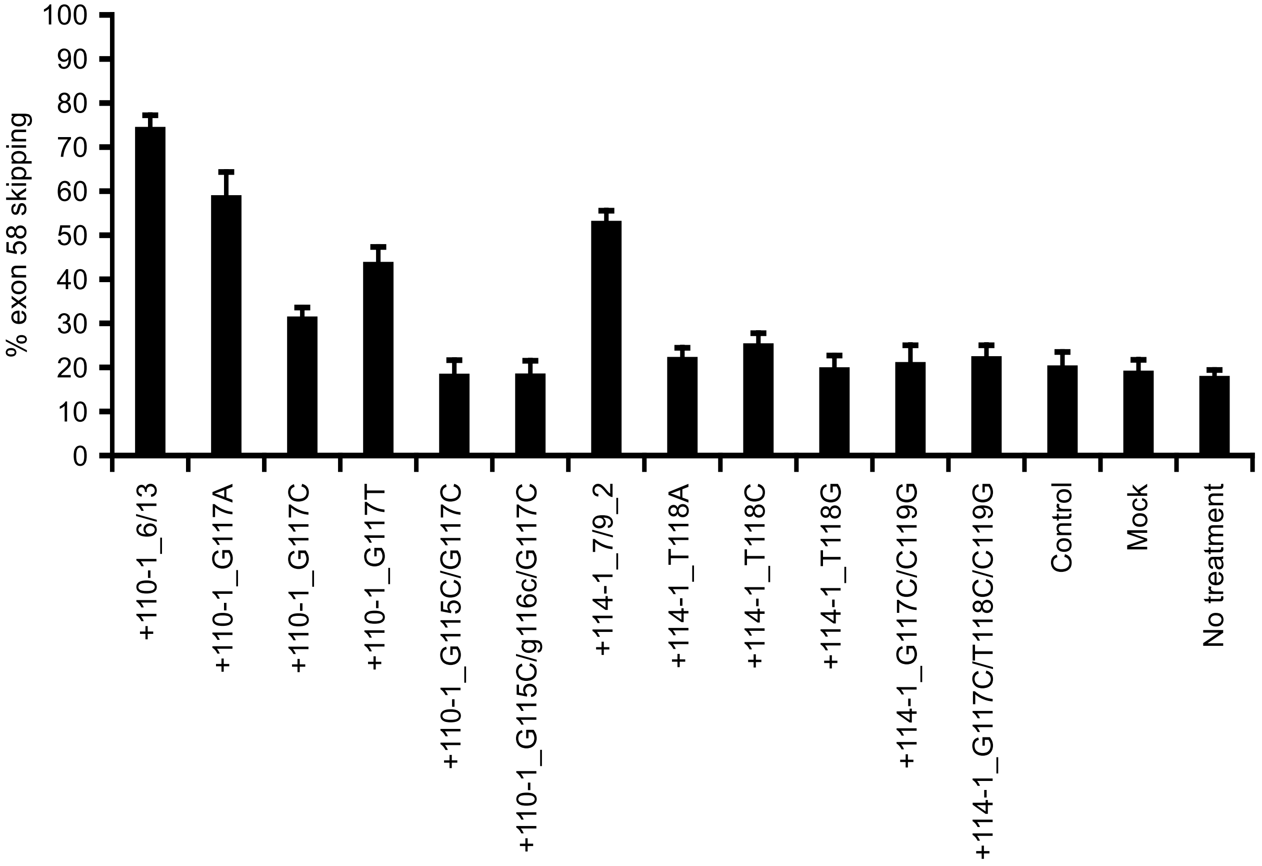

Supplementary Fig. 11

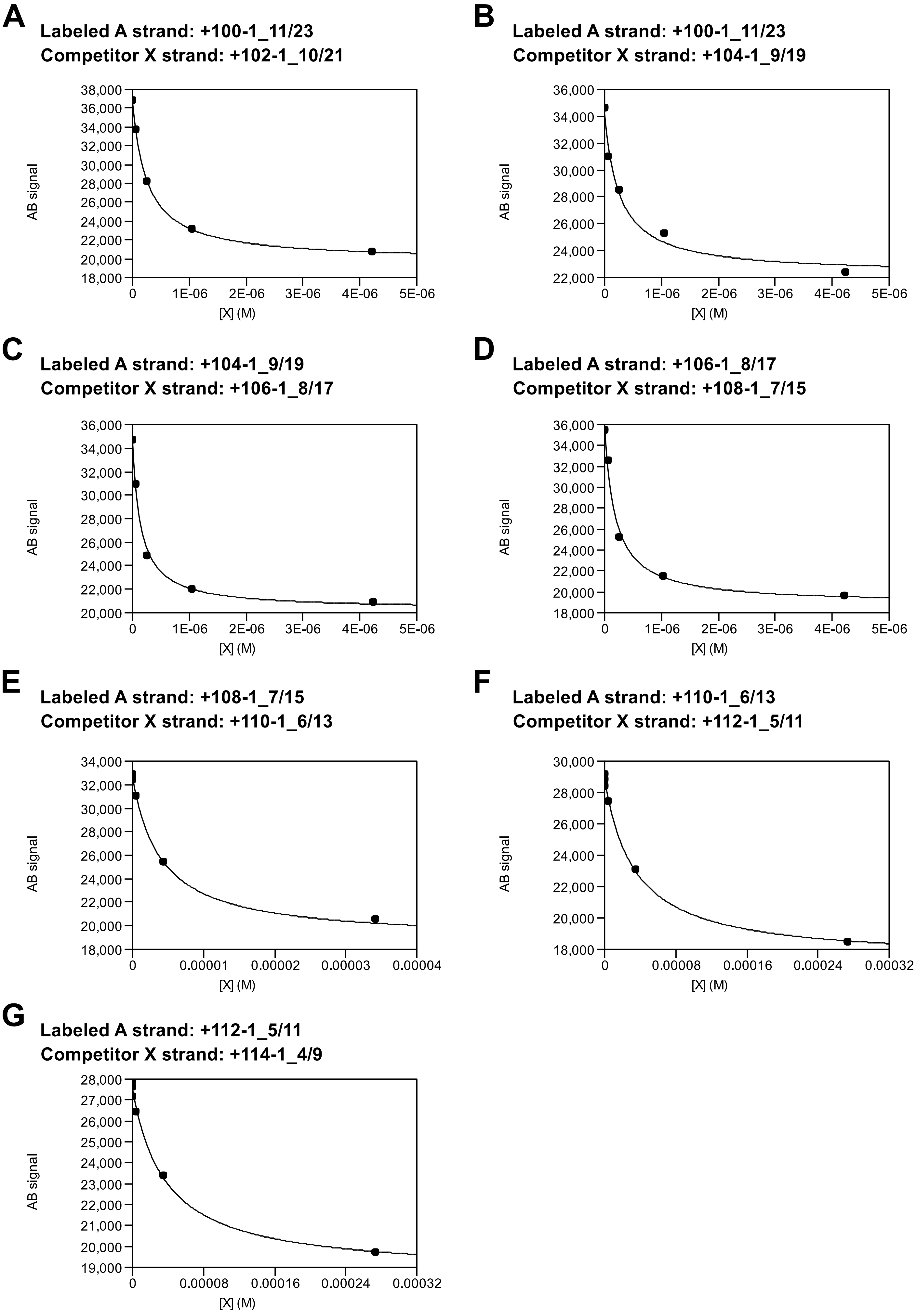

Supplement: SUPPLEMENTARY DATA [file supp_gku512_nar-03125-f-2013-File010.pdf]
